# Mitogenomic Insights into the Hampala Barb (*Hampala macrolepidota*) from Sumatra, Indonesia: Characterization, Phylogenetic Placement, and Genetic Diversity

**DOI:** 10.3390/biom16020185

**Published:** 2026-01-26

**Authors:** Arief Wujdi, Angkasa Putra, Sarifah Aini, Gyurim Bang, Yunji Go, Ah Ran Kim, Soo Rin Lee, Kyoungmi Kang, Hyun-Woo Kim, Shantanu Kundu

**Affiliations:** 1 Interdisciplinary Program of Marine and Fisheries Sciences and Convergent Technology, Pukyong National University, Busan 48513, Republic of Korea; 2 Research Organization for Earth Sciences and Maritime, National Research and Innovation Agency (BRIN), Jakarta 10340, Indonesia; 3 Industry 4.0 Convergence Bionics Engineering, Pukyong National University, Busan 48513, Republic of Korea; 4 Marine Integrated Biomedical Technology Center, National Key Research Institutes in Universities, Pukyong National University, Busan 48513, Republic of Korea; 5 Research Center for Marine Integrated Bionics Technology, Pukyong National University, Busan 48513, Republic of Korea; 6 Ocean and Fisheries Development International Cooperation Institute, College of Fisheries Science, Pukyong National University, Busan 48513, Republic of Korea; 7 Department of Marine Biology, Pukyong National University, Busan 48513, Republic of Korea; 8 Department of Biology, Faculty of Science and Technology, Airlangga University, Surabaya 60115, Indonesia; 9 International Graduate Program of Fisheries Science, Pukyong National University, Busan 48513, Republic of Korea

**Keywords:** biodiversity hotspots, freshwater fish, matrilineal phylogeny, mitochondrial DNA, population structure, Southeast Asia

## Abstract

Despite its ecological and economic importance, *Hampala macrolepidota* (Cyprinidae: Smiliogastrinae) remains taxonomically debated, having undergone historical reclassifications across multiple taxonomic ranks. These challenges highlight the urgent need for integrative genomic analyses to resolve its phylogeny and assess genome-wide diversity, establishing a baseline for effective management and conservation. In this study, the newly assembled mitogenome of *H. macrolepidota* from within its native range in Lake Dibawah, West Sumatra, Indonesia, was sequenced. The mitogenome spanned 17,104 bp, encoded 37 genes and a control region, and exhibited a nucleotide composition biased toward adenine and thymine. The protein-coding genes (PCGs) predominantly utilized ATG as the initiation codon and showed a higher proportion of hydrophobic compared to hydrophilic amino acids. The nonsynonymous (Ka) and synonymous (Ks) substitution ratios were below ‘1’, which indicates negative selection on most of the PCGs within *Hampala* and other Smiliogastrinae species. Mitogenome-wide analysis revealed overall high intraspecific genetic diversity (≥2.7%) in the native Indonesian population compared to mainland populations in Southeast Asia. The Bayesian and maximum-likelihood phylogenetic analyses elucidated matrilineal evolutionary relationships within the subfamily Smiliogastrinae, with the *Hampala* species forming a monophyletic cluster. The present mitogenome-based phylogenetic topologies also supported the taxonomic placement of several species in the revised classification, which previously were classified under the genera *Puntius* and *Barbus*, respectively. Additionally, the investigation of partial mitochondrial *COI* and *Cytb* genes further elucidated the population genetic structure of *H. macrolepidota* across Southeast and East Asia. The observed genetic divergence (0–4.2% in *COI* and 0–4.5% in *Cytb*), together with well-resolved phylogenetic clustering and the presence of both shared and distinct haplotypes among Indonesian samples, provides strong evidence for long-term population isolation and local adaptation. These patterns are most plausibly driven by historical hydrological dynamics, paleo-drainage connectivity, and persistent geographic barriers that have structured population divergence over time. In addition, this study emphasizes the need to generate mitogenomes of seven additional *Hampala* species from Southeast Asia to better understand their evolutionary patterns. Further, broader sampling of wild *H. macrolepidota* populations across their biogeographical range will be essential to strengthen understanding of their genetic diversity and guide effective conservation strategies.

## 1. Introduction

Mitochondrial genome studies have emerged as essential tools in systematic and taxonomic research in recent decades, providing significant insights into the genetic diversity and evolutionary relationships among diverse organisms [1,2]. In vertebrates, the mitogenome typically comprises a circular DNA molecule spanning approximately 16–17 kilobase pairs in length, comprising 13 protein-coding genes (PCGs), 2 ribosomal RNA (rRNA) genes, 22 transfer RNA (tRNA) genes, and a noncoding control region (CR) [3,4]. The mitochondrial genes are highly conserved in sequence length and base composition across taxa, and exhibit distinctive characteristics such as maternal inheritance, compact genome size, elevated mutation rates, lack of recombination, and high cellular copy numbers [5,6]. These attributes make mitogenomes particularly valuable for species characterization through structural and sequence-variation analyses [5,7]. Compared to the fragmented partial genes from both mitochondrial and nuclear genes, complete mitogenomes offer substantial advantages for elucidating evolutionary patterns, particularly in ichthyology studies [7,8]. The use of mitogenomes has demonstrated robust confidence in species classification by matrilineal phylogenetic tree reconstruction for an extensive group of marine and freshwater fishes, particularly highlighting the most biodiverse freshwater fish group, cyprinids [5,9]. Despite the swift global advancement of this genomic method, publicly available reference mitogenomic data for numerous fish species continue to be inadequately represented [4,10]. Thus, expanding mitogenomic study across fish taxa is critical to strengthening phylogenetic resolution, biogeographic interpretation, and evidence-based conservation strategies.

The family Cyprinidae (order Cypriniformes) is the largest family of freshwater fishes, comprising 1802 extant valid species divided into 168 valid genera dispersed widely across North America, Africa, and Eurasia [11]. In Southeast Asia, cyprinids inhabit a variety of habitats, including streams, springs, lakes, and swamps, with certain species found solely in subterranean ecosystems [12]. The extensive variety of environments inhabited by the cyprinid species has resulted in a corresponding diversity of adaptations in ecology, morphology, behavior, coloration, and other traits [13]. Moreover, cyprinid fishes exhibit strong adaptive capacity, resulting in the emergence of either novel or convergent characters across different taxonomic hierarchies [14]. Consequently, the systematics and taxonomy of cyprinids have always been subject to controversy and are likely to undergo revisions over time, with 231 species under 27 genera newly described in the past decade, underscoring ongoing systematic efforts to understand the remarkable diversity of this group [11]. The genus *Hampala* (subfamily Smiliogastrinae) contains 10 valid species distributed in both the mainland and islands of Southeast Asia [11]. The Hampala barb, *Hampala macrolepidota*, is distributed across the Mekong and Chao Phraya River basins, as well as in Brunei, Malaysia (Peninsular, Sarawak, and Sabah), and Indonesia (Borneo, Sumatra, and Java), according to assessments by the IUCN Freshwater Fish Specialist Group (FFSG) [15]. Earlier studies have also reported the presence of *H. macrolepidota* in Mengla, Yunnan Province, Southwest China, while its occurrences in Hong Kong are likely the result of introductions via the aquarium trade and aquaculture practices rather than representing native populations [16,17,18]. This cyprinid fish is carnivorous and is very sensitive to poor water quality; therefore, it is often utilized as a suitable bio-indicator species for evaluating contamination by potentially harmful elements in aquatic environment [19,20]. Furthermore, owing to its economic market value and ecological significance, *H. macrolepidota* is susceptible to extensive exploitation due to anthropogenic pressure [21,22]. Therefore, a comprehensive approach combining morphology and molecular analyses is essential for precise identification, in-depth molecular characterization, and phylogenetic placement of this species, which are critical components for establishing more effective fishery management and conservation practices.

Numerous studies have employed morphology-based inference to investigate the population dynamics, growth, and utilization of *H. macrolepidota* [21,22,23,24]. Moreover, molecular investigations using various partial mitochondrial and nuclear gene markers successfully revealed genetic characterization, population genetic diversity, and phylogenetic placement of *H. macrolepidota* along with its congeners [17,25,26,27,28]. Molecular techniques also facilitate high-throughput DNA sequencing to identify *H. macrolepidota* and other cyprinids from environmental samples [29]. However, the taxonomic classification of *H. macrolepidota* has remained phylogenetically contentious for over a decade, with uncertainty across multiple taxonomic ranks. More specifically, previous phylogenetic inference using a combination of partial mitochondrial genes (*Cytb*, *16S rRNA*, and *D-loop*) as well as a nuclear gene (*RAG2*) classified *H. macrolepidota* within the Barbinae subfamily [16,30,31,32]. In contrast, subsequent studies placed *H. macrolepidota* in the Cyprininae subfamily based on the use of both *RAG1* and *Cytb* genes, as well as integrated use of mito-nuclear genes and pharyngeal teeth structure [17,33]. In addition, based on nucleo-mitochondrial-gene-based phylogenetic analyses, the *Hampala* species were placed within the tribe Smiliogastrini, which was subsequently elevated to become a distinct subfamily, Smiliogastrinae [34,35]. However, the precise phylogenetic placement of the *Hampala* species within the subfamily Smiliogastrinae has never been rigorously tested and requires critical investigation using genomic data, especially for samples within their native range. Prior to this study, the mitogenome of *H. macrolepidota* was generated from unknown localities, providing information on gene organization and boundaries; however, the phylogenetic reconstruction is still underrepresented [36]. Hence, this study aimed to generate a newly sequenced mitogenome of *H. macrolepidota* from its native range in Lake Dibawah, West Sumatra, Indonesia. Additionally, the study provided comparative analyses to assess the structural and sequence variation with two other *Hampala* species (*H. dispar* and *H. salweenensis*). By using mitogenomic data, this study also investigates the maternal phylogenetic relationships of *Hampala* and other closely related species within the subfamily Smiliogastrinae. The study further offers valuable insights into the mitogenome-wide population genetic structure of *H. macrolepidota* between its native population in Indonesia in comparison with prior generated data possibly sourced from the mainland of Southeast Asia. The findings from this study assess the existing taxonomic classification framework of the *Hampala* species and enhance our understanding of the genetic diversity of this cyprinid in Southeast and East Asia.

## 2. Materials and Methods

### 2.1. Sample Collection and Species Identification

**Figure 1:**
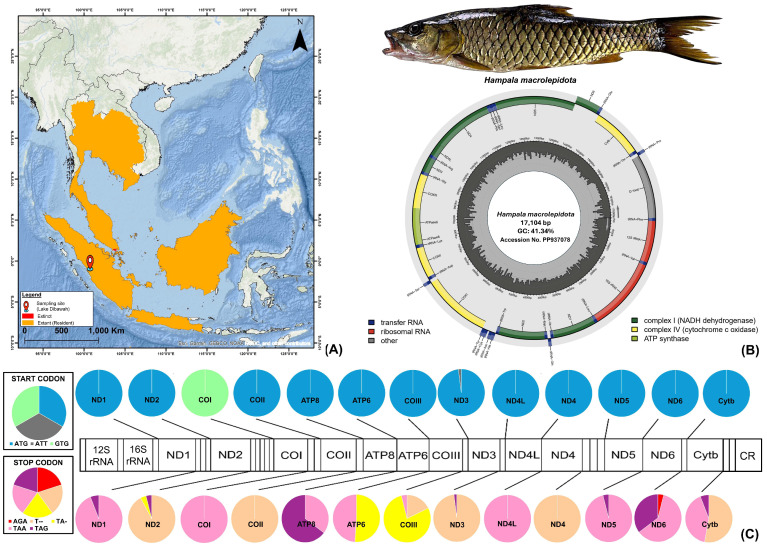
(**A**) The biogeographic distribution of *H. macrolepidota* across Southeast Asia based on the presence of extant (resident) populations as well as extinct populations based on the IUCN Red List of Threatened Species [15]. The sampling location of *H. macrolepidota* is marked with a red pin. (**B**) The mitogenome of *H. macrolepidota* (PP937078), sampled from Lake Dibawah, West Sumatra, Indonesia, was annotated using the MitoAnnotator tool [4]. The colored arcs indicate the positions of various genes, including PCGs, rRNAs, tRNAs, and CR. The species photograph was taken by Hamdani from Jakarta Technical University of Fisheries, Indonesia. (**C**) The usage frequencies of start and stop codons in the 13 PCGs of *H. macrolepidota* and other congeneric species within the subfamily Smiliogastrinae.

A wild specimen of *H. macrolepidota* was collected from Lake Dibawah, West Sumatra, Indonesia (1.021111° S, 100.725° E) (Figure 1A) using a 1.59 cm mesh gill net. Species identification was performed using key morphological characters [37,38], including orange-to-brown caudal-fin colorization with a black longitudinal stripe and 24–25 scales on the lateral line. The specimen measured 25.8 cm in total length and approximately 200 g in body weight. The muscle tissue was aseptically collected from the epaxial region between the dorsal fin and lateral line. The sample was immediately placed in a 2 mL tube, preserved in 95% ethanol, and stored at −20 °C to minimize DNA degradation and cross-contamination. The voucher specimen was fixed in 10% neutral buffered formaldehyde and archived in the Jakarta Technical University of Fisheries, Pariaman Campus, West Sumatra Province, Indonesia (voucher code IDN8). For further laboratory examination by molecular analysis, the tissue sample was sent to the Molecular Physiology Laboratory at Pukyong National University, Busan, South Korea. The species examined in this study is commonly captured by local fishermen and consumed by surrounding communities. It is not listed as a protected fish species in Indonesia and is classified as ‘Least Concern’ on the IUCN Red List of Threatened Species (https://www.iucnredlist.org/). Nevertheless, all study design and sampling procedures were reviewed and approved by the Pukyong National University Institutional Animal Care and Use Committee (IACUC) under approval no. PKNUIACUC-2025-16 (dated 18 February 2025) and were conducted in accordance with the ARRIVE 2.0 guidelines for animal research (https://arriveguidelines.org/) [39].

### 2.2. DNA Extraction and COI Marker Sequencing

DNA extraction was performed using the AccuPrep^®^ Genomic DNA Extraction Kit (Bioneer, Daejeon, Republic of Korea) following the manufacturer’s standard guidelines. In brief, 30 mg of muscle tissue was digested with proteinase K and sodium dodecyl sulfate (SDS) to lyse the cells and degrade proteins, followed by column-based washing and purification with binding buffers. The purified DNA was eluted in 50 μL of TE buffer, followed by DNA-concentration quantity checking using a NanoDrop^TM^ spectrophotometer (Thermo Fisher Scientific, Waltham, MA, USA). Polymerase chain reaction (PCR) was performed in 30 μL reactions using a Takara thermal cycler with the following components: 3 μL 10× ExTaq Buffer, 3 μL dNTPs, 1 μL each of forward and reverse primer set, 0.2 μL Ex Taq HS DNA polymerase enzyme (Takara Korea Biomedical, Inc., Seoul, Republic of Korea), 0.9 μL dimethyl sulfoxide DMSO (3%), 1 μL of 1/10 diluted template of DNA, and 19.9 μL molecular-grade water. A paired-forward Fish-BCL (5′-ACTTCYGGGTGRCCRAARAATCA-3′) and reverse Fish-BCH (5′-TCAACYAATCAYAAAGATATYGGCAC-3′) primer set was utilized to amplify the targeted ~650 bp of barcoding area in the mitochondrial *COI* gene marker [40]. The thermal cycling conditions consisted of initial denaturation at 94 °C for 3 min; 40 cycles of 94 °C for 30 s, 50 °C for 30 s, and 72 °C for 1 min; followed by final extension at 72 °C for 5 min. The PCR products were purified with the AccuPrep^®^ PCR/Gel Purification Kit (Bioneer, Daejeon, Republic of Korea), and subjected to bidirectional Sanger sequencing on a capillary sequencer (Macrogen, Inc., Daejeon, Republic of Korea). The resulting raw chromatogram sequences were quality-filtered using SeqScanner v1.0 (Applied Biosystems, Inc., Foster City, CA, USA). The *COI* sequence obtained was examined for species determination using nucleotide BLAST search (https://blast.ncbi.nlm.nih.gov/Blast.cgi, accessed on 25 October 2025) and was submitted to the GenBank database (accession no. PX107884).

### 2.3. Mitogenome Sequencing and Annotation

The complete mitogenome of *H. macrolepidota* was sequenced using the next-generation sequencing (NGS) platform with 2 × 150 bp paired-end reads using Illumina NovaSeq (Illumina, Inc., San Diego, CA, USA) at Macrogen (Daejeon, Republic of Korea; https://dna.macrogen.com/). The library preparation utilized the TruSeq Nano DNA High-Throughput Library Prep Kit following the manufacturer’s protocol (Illumina, Inc., San Diego, CA, USA). In brief, 100 ng of genomic DNA was fragmented using Covaris M220 adaptive focused-ultrasonication (Covaris, Woburn, MA, USA). Fragments were end-repaired, 5′-phosphorylated, and A-tailed before ligation with dual-indexed TruSeq UD adapters (Illumina, Inc., San Diego, CA, USA). The size selection was performed using the AMPure XP magnetic-bead-based technique followed by eight cycles of PCR enrichment. The library quality was assessed by qPCR following the standard Illumina sequencing protocol using the KAPA Library Quantification Kit, and quality assessment was accomplished using a 4200 TapeStation D1000 (Agilent Technologies, Santa Clara, CA, USA). The high-quality paired-end reads generated by NGS, totaling 15,255,172 bp, resulted in an average sequencing depth of approximately 100×. The reads were assembled using Geneious Prime v11.0.2 [41] and compared to the previous mitogenome sequence of *H. macrolepidota* (accession no. KF670818) [36]. The final assembly yielded a single circular mitochondrial contig with a contig N50 value of 17,128 bp. For precise sequence assembly, the overlapping regions were aligned using MEGA v11 [42]. The gene’s organization and boundaries were annotated using the Mitofish MitoAnnotator web server (https://mitofish.aori.u-tokyo.ac.jp/annotation/input/, accessed on 25 October 2025) [4] and MITOS de novo annotation of metazoan mitochondrial genomes in the Galaxy v1.1.6 web server (https://usegalaxy.eu) [43]. Moreover, the start and stop codons of PCGs were verified using the Open Reading Frames (ORFs) Finder tool in GenBank (https://www.ncbi.nlm.nih.gov/orffinder, accessed on 25 October 2025). The final annotated *H. macrolepidota* mitogenome was submitted to the GenBank database (accession no. PP937078).

### 2.4. Mitogenome Characterization and Comparative Analyses

The comparative analyses were conducted between the newly sequenced *H.* macrolepidota from Indonesia and published conspecific sequences (accession nos. KF670818 and AP011186), with particular focus on structural variations among mitogenomes. The intergenic spaces and overlap between contiguous genes were tabulated manually in Microsoft Excel v2016. Base compositions of all mitochondrial genes were calculated using MEGA v11, while A-T and G-C skewness was calculated using a formula established in a previous study [44]. The nucleotide diversity (π) among *Hampala* species was assessed according to the sliding-window method in DnaSP v6.0 with a window size of 200 bp and a step size of 25 bp [45]. The implementation of these fragment sizes enhances prediction accuracy for eukaryotic PCG boundaries [46]. The pairwise mutation rates assessed through identification of nonsynonymous (Ka) and synonymous (Ks) substitutions of *H. macolepidota* and its congeneric species within the Smiliogastrinae were calculated using DnaSP v6.0. In addition, substitution saturation analysis was performed on all Smiliogastrinae PCGs to quantify transition and transversion rates using DAMBE v6 [47]. The Relative Synonymous Codon Usage (RSCU), amino acids composition, and codon distribution per thousand codons (CDsPT) were analyzed by aligning the PCGs of all *Hampala* species in MEGA v11. The secondary structures of tRNAs were predicted using the ARAGORN function in the Galaxy web server [48]. Conserved domains were identified by aligning all CRs of the *Hampala* species using CLUSTAL X alignment in MEGA v11 referring to previously published sequences for cyprinids [5,49]. The CR sequence repetitions were detected using the Tandem Repeats Finder web server (https://tandem.bu.edu/trf/trf.html, accessed on 25 October 2025) [50].

### 2.5. Dataset Construction and Phylogenetic Inferences

The phylogenetic analyses encompassed 48 species’ mitogenomes from the subfamily Smiliogastrinae, incorporating the newly sequenced *H. macrolepidota* mitogenome (Table S1). To reconstruct matrilineal phylogeny within *Hampala* and related smiliogastrin taxa, we generated a concatenated alignment of all 13 PCGs of representative mitogenomes using the concatenator module in iTaxoTools v0.1 [51]. In addition to the generated sequences, a total of 44 partial *COI* and 112 *Cytb* sequences of *H. macrolepidota* were retrieved from GenBank (Table S2). The phylogenetic reconstructions based on the mitogenome as well as partial mitochondrial genes datasets employed the dark mahseer, *Naziritor chelynoides*, from the subfamily Torinae (accession no. PP894694), as the outgroup [52]. Model selection using PartitionFinder v2 [53] identified ‘GTR + R’ and ‘GTR + G + I’ as the best-fit substitution models for the mitogenome and partial-gene datasets, respectively, based on the lowest Bayesian Information Criterion (BIC) values [54]. Bayesian (BA) phylogenetic inference was performed for both mitogenomic and partial-gene datasets using MrBayes v3.1.2 [55], with a Metropolis-coupled Markov Chain Monte Carlo (MCMC) algorithm with *nst* = 6. The BA tree was constructed through 1,000,000 generations, with trees sampled every 100 generations, and the first 25% of samples were discarded as burn-in. Moreover, a maximum-likelihood (ML) phylogenetic tree was constructed exclusively using the mitogenomic dataset in the PhyML v3.0 web server (http://www.atgc-montpellier.fr/phyml/, accessed on 25 October 2025) [56]. All phylogenetic trees were exported to Newick format and subsequently revisualized using the Interactive Tree of Life (iTOL) v7 web server (https://itol.embl.de/login.cgi, accessed on 25 October 2025), enabling advanced customization of tree topology [57].

### 2.6. Genetic Distance and Population Structure

To investigate the intra- and inter-species genetic distances, a concatenated dataset of 13 PCGs of three *Hampala* species (five sequences) was analyzed using the Kimura 2-parameter (K2P) model in MEGA v11. Moreover, to examine finer-scale variation in genetic distances and variable sites at the intra-species level in *H. macrolepidota* from Sumatra, Indonesia, each PCG was analyzed separately, including two previously generated mitogenomes (accession nos. KF670818 and AP011186) most likely from the mainland of Southeast Asia. In addition, the intra-species genetic diversity and population structure of *H. macrolepidota* were evaluated using both *COI* (*n* = 45) and *Cytb* (*n* = 113) gene sequences (Table S2). The genetic diversity parameters, such as haplotype diversity (Hd), number of haplotypes, and nucleotide diversity (π), were estimated using DnaSP v6.0. Subsequently, the haplotype networks of *H. macrolepidota* population were constructed using POPART v.1.7 software with the Templeton, Crandall, and Sing (TCS) algorithm [58,59], employing three datasets, including 13 concatenated PCGs, *COI*, and *Cytb* genes. The population clusters were delineated based on mutational step counts, thereby elucidating the phylogeographic structure of *H. macrolepidota* in Southeast and East Asia.

## 3. Results

### 3.1. Mitogenome Structure and Gene Organization

The newly assembled circular mitogenome of *H. macrolepidota* (GenBank accession no. PP937078) comprises 17,104 bp, containing the standard vertebrate complement of 37 genes, including 13 PCGs, 2 rRNAs, 22 tRNAs, and 1 CR. The gene distribution exhibited the typical heavy-strand bias, with 28 genes (12 PCGs, 14 tRNAs, and both rRNAs) encoded on the heavy strand, while the remaining 9 genes (1 PCG and 8 tRNAs) resided on the light strand (Figure 1B, Table 1). The comparative mitogenomic analysis across *Hampala* species revealed size variation ranging from 15,635 bp for *H. dispar* (without CR) to 17,120 bp for the *H. macrolepidota* sequence (AP011186), while maintaining consistency on strand-specific gene distribution patterns across all *Hampala* species. Moreover, the mitogenome of *H. macrolepidota* showed a bias toward adenine-thymine (AT) composition (58.66%), comprising 33.69% for adenine (A), 24.97% for thymine (T), 14.80% for guanine (G), and 26.54% for cytosine (C). A similar AT bias in nucleotide composition was observed in mitogenomes from other *Hampala* species, with values ranging from 53.95% in *H. dispar* to 58.96% in *H. salweenensis*. The AT skew and GC skew values in the newly sequenced *H. macrolepidota* mitogenome were determined as 0.149 and −0.284, respectively. Comparative analysis across all *Hampala* mitogenomes showed that AT skew values varied from 0.140 in *H. salweenensis* to 0.151 in *H. macrolepidota* (KF670818), whereas GC skew values varied from −0.285 in *H. macrolepidota* (KF670818) to −0.326 in *H. macrolepidota* (AP011186) (Table 2). Furthermore, the newly sequenced mitogenome structure of *H. macrolepidota* showed five overlapping coding regions, spanning 22 bp, with the greatest overlap occurring between *ATP8* and *ATP6*, as well as *ND4L* and *ND4*, each comprising 7 bp. The mitogenome of *H. macrolepidota* also showed 16 intergenic spaces totaling 68 bp, with the largest spacer (35 bp) situated between *tRNA-Asn* and *tRNA-Cys*. Comparative analysis with other *Hampala* mitogenomes illustrated that the lengths of overlapping coding regions varied from 5 bp to 6 bp, with the longest overlap (7 bp) occurring consistently in all *Hampala* species between *ATP8* and *ATP6*, as well as *ND4L* and *ND4* genes. The lengths of intergenic spacers ranged from 13 bp to 16 bp, with the largest spacer (35 bp) consistently observed between *tRNA-Asn* and *tRNA-Cys* (Table S3).

### 3.2. Protein-Coding Gene Features

**Figure 2:**
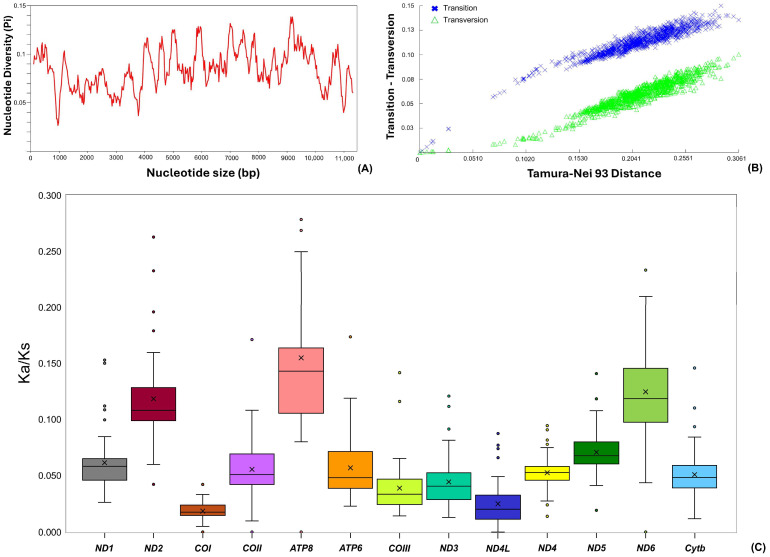
(**A**) Nucleotide diversity (π) for mitochondrial PCGs across various species within the subfamily Smiliogastrinae. (**B**) Relationship between transitions (s) and transversions (v) and genetic divergence in PCGs, based on TN93 distances. (**C**) Pairwise divergence of Ka/Ks ratios for each PCG of *H. macrolepidota* and other cyprinids under the subfamily Smiliogastrinae.

The newly sequenced mitogenome of *H. macrolepidota* in this study consisted of 13 PCGs with a total length of 11,045 bp, contributing 66.7% of the total mitogenome length. Among these PCGs, the shortest length was observed in the *ATP8* gene (165 bp), while the longest gene was *ND5* with 1824 bp (Table 1). The total length of PCGs in other *Hampala* mitogenomes varied from 11,401 bp in *H. salweenensis* to 11,407 bp in *H. macrolepidota* (AP011186). The PCGs of the newly sequenced *H. macrolepidota* exhibited an AT bias of 58.18%. The bias toward AT composition reflected an asymmetry in strand-specific nucleotide composition, yielding an AT skew of 0.126 and GC skew of −0.354. The comparative analysis showed similarity in AT bias in other *Hampala* mitogenomes, with the value ranging from 58.20% in *H. macrolepidota* (AP011186) to 58.84% in *H. salweenensis*. The AT skewness value ranged from 0.064 in *H. salweenensis* to 0.073 in *H. macrolepidota* (AP011186 and KF670818) and the GC skewness values were from −0.308 in *H. macrolepidota* (AP011186) to −0.301 in *H. dispar* (Table 2). Among the 13 PCGs in *H. macrolepidota*, 12 utilized ATG as the start codon, while *COI* uniquely employed GTG. In terms of termination codon analysis, five PCGs were terminated by complete TAA stop codons, two genes (*ATP8* and *ND6*) terminated with TAG, and six PCGs exhibited incomplete stop codons (TA- or T--). Comparative analysis in 13 PCGs of 48 species (50 sequences) under the subfamily Smiliogastrinae exhibited three types of start codon (Figure 1C). ATG served predominantly in 12 PCGs for almost all species, except *ND3* of *H. salweenensis* which utilized ATT as start codon. The consistent use of GTG as a start codon was found exclusively for *COI* for all species examined. In terms of termination codon, the TAA type was the most frequently found in major PCGs, followed by the incomplete T-- codon which terminated *COII*, *COIII*, *Cytb*, *ND2*, *ND3*, and *ND4* across all Smiliogastrinae species (Figure 1C, Table S4). Further analysis of nucleotide diversity on PCGs showed the average value of nucleotide diversity (π) was 0.084, with *ND5* exhibiting the highest value of 0.138 across all the *Hampala* species (Figure 2A). In addition, Tamura–Nei (TN93) divergence values calculated across all PCGs of the mitogenomes from 48 fish species within the subfamily Smiliogastrinae indicated that neither transitions nor transversions had reached saturation (Figure 2B).

### 3.3. Substitution Pattern and Relative Synonymous Codon Usage

**Figure 3:**
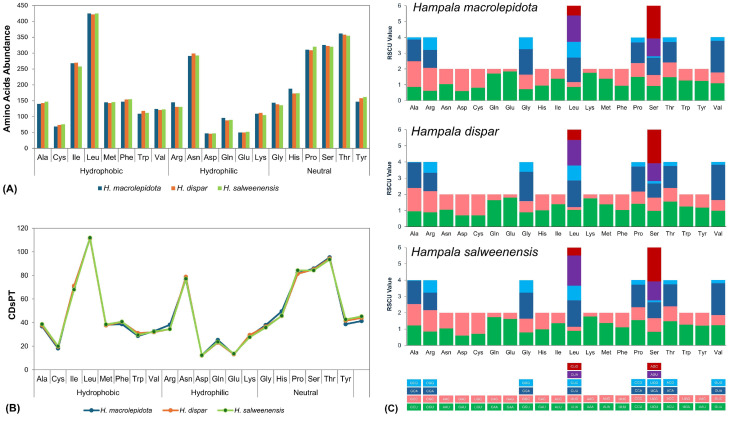
Structural characteristics of amino acids in PCGs across three *Hampala* species: (**A**) Comparative analysis of amino acid composition. (**B**) Codon distribution per thousand codons for all amino acids in mitogenomes. (**C**) The RSCU analysis, highlighting codon preferences contributing to the translational efficiency of each amino acid.

The nonsynonymous (Ka) and synonymous (Ks) substitution ratios showed that all PCGs of *H. macrolepidota* and its congeneric species in the subfamily Smiliogastrinae experienced a similar pattern of natural selection pressure. Overall, the Ka/Ks ratio value highlighted ratios below ‘1’ across all the Smiliogastrinae subfamily members. The mean Ka/Ks values ranged from 0.018 ± 0.008 for *COI* to 0.156 ± 0.094 for *ATP8*. Subsequently, the Ka/Ks ratios across all Smiliogastrinae species in the current dataset follow the order *COI* < *ND4L* < *COIII* < *ND3* < *Cytb* < *ND4* < *COII* < *ATP6* < *ND1* < *ND5* < *ND2* < *ND6* < *ATP8* (Figure 2C, Table S5). Analysis of RSCU indicated that a total of 3643 amino acids were translated from the 13 PCGs of *H. macrolepidota*. The composition of amino acids demonstrated a predominant contribution of leucine (11.7%), threonine (9.9%), and serine (8.9%), while cystine (1.9%), glutamic acid (1.4%), and aspartic acid (1.3%) exhibited a lesser abundance (Figure 3A, Table S6). The comparative analysis illustrated consistent patterns in amino acid composition across *Hampala* species, with similar prevalence of leucine, threonine, and serine. The abundance of leucine in the *Hampala* species was also associated with the highest CDsPT value for leucine, ranging from 111.11 in *H. dispar* to 111.87 in *H. salweenensis*. Conversely, the low abundance of aspartic acid was supported with the lowest CDsPT value, ranging from 12.11 in *H. dispar* to 12.37 in *H. salweenensis* and *H. macrolepidota* (Figure 3B, Table S7). The predominance of leucine and serine was supported by six codon variations across all *Hampala* species (Figure 3C). Moreover, several codons displayed higher relative usage compared to the other codons, indicated by values greater than 1.5, reflecting their preferential contribution for amino acid translation. Notably, AGC for serine, as well as CTA and CTT for leucine, showed elevated usage in all *Hampala* species (Table S6).

### 3.4. Ribosomal RNA and Transfer RNA Structures

The newly sequenced mitogenome of *H. macrolepidota* contained 954 bp 12S rRNA (small subunit) and 1675 bp 16S rRNA (large subunit) genes, collectively spanning 2629 bp and contributing to 15.37% of total mitogenome length (Table 1). The comparative analysis revealed that the total rRNA length ranged from 2627 bp in *H. salweenensis* to 2631 bp in *H. dispar* and *H. macrolepidota* (AP011186 and KF670818). The rRNA genes exhibited a bias toward AT, with proportions ranging from 54.62% in *H. macrolepidota* (AP011186) to 55.83% in *H. dispar*. The AT skewness varied from 0.284 in *H. dispar* to 0.308 in *H. macrolepidota* (KF670818), while the GC skewness values ranged from −0.110 in *H. macrolepidota* (KF670818) to −0.093 in *H. dispar* (Table 2). Furthermore, the *H. macrolepidota* mitogenome contained 22 tRNAs interspersed between rRNA and PCGs, with a combined length of 1561 bp contributing 9.13% of total genome size (Table 1). All tRNAs exhibited significant AT (55.89%) asymmetry composition, reflected in calculated skewness values of 0.132 for AT skew and −0.149 for GC skew, respectively (Table 2). Additionally, uniformity in the anticodon sequences of all 22 tRNAs was observed across *Hampala* species (Table S8). Secondary structure prediction revealed that 21 of the 22 tRNAs adopt the conventional cloverleaf conformation, while *tRNA-Ser1* lacks the dihydrouracil (DHU) arm due to missing nucleotide bonds. Among these, 16 tRNAs incorporated both Watson–Crick base pairing (A=T, G≡C) and non-canonical base pairings (G-T, T-T) in their stems, whereas the remaining 6 tRNAs exhibited Watson–Crick base pairing exclusively (Figure S1).

### 3.5. Control Region Architectures

**Figure 4:**
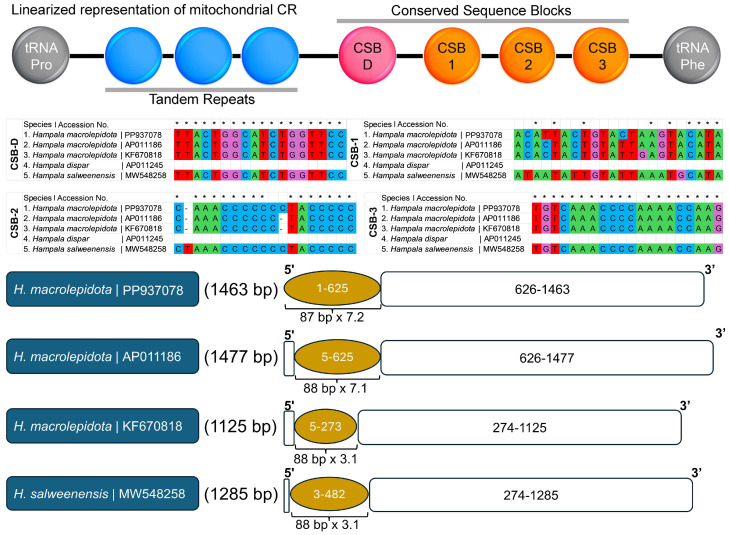
Nucleotide features of CRs in the mitogenomes of three *Hampala* species, emphasizing the conserved domains and their lengths. Highly conserved nucleotides are denoted by black asterisks. The panel below depicts the presence of tandem repeats, along with their sequence characteristics in two *Hampala* species.

The CR length of *H. macrolepidota* was 1463 bp, contributing 8.5% of the total mitogenome length. The CR exhibited an AT bias of 71.84%, resulting in AT and GC skewness values of 0.087 and −0.199, respectively (Table 1). Comparative analysis among different *Hampala* mitogenomes revealed variation in CR length, ranging from 1125 bp in *H. macrolepidota* (KF670818) to 1477 bp in *H. macrolepidota* (AP011186). Notably, the CR of all *Hampala* species also showed a similar bias toward AT content, and AT skewness varied from 0.082 in *H. salweenensis* to 0.101 in *H. macrolepidota* (KF670818). Since the CR showed a bias towards AT, the GC content was lower, resulting in GC skewness ranging from −0.244 in *H. salweenensis* to −0.151 in *H. macrolepidota* (AP011186) (Table 2). Due to the inability to identify the CR sequence in the incomplete *H. dispar* mitogenome, comparative analyses of nucleotide composition and other CR-related features were performed only for the remaining two *Hampala* species. Notably, the five CR sequences of *Hampala* also incorporated four conserved sequence blocks (CSBs): CSB-1, CSB-2, CSB-3, and CSB-D. Among all conserved domains, CSB-1 was the largest (21 bp), followed by CSB-3 and CSB-2 (19 bp each) and CSB-D (18 bp). In regards of CSB-D and CSB-3, all *Hampala* mitogenome sequences showed a similar conserved nucleotides pattern. In CSB-1, all *Hampala* mitogenome sequences displayed major variation, sharing only nine conserved nucleotides of the total length (21 bp). In CSB-2, the previously sequenced *H. macrolepitoda* (AP011186 and KF670818) showed a consistent pattern in conserved nucleotides and it had 1 bp differences compared to the newly sequenced mitogenome of *H. macrolepidota* from this study. Moreover, all the CRs of the *Hampala* species demonstrated long repetitive nucleotide patterns in the extended termination-associated sequences (ETAS) region. The newly sequenced mitogenome of *H. macrolepidota* comprised seven tandem copies of 87 bp consensus nucleotides. In comparison, other sequences of *H. macrolepidota* (AP011186 and KF670818) possessed seven and three copies of 88 bp sequences, respectively, while *H. salweenensis* contained three copies of 88 bp tandem repeat nucleotides (Figure 4).

### 3.6. Matrilineal Phylogenetic Relationships of Smiliogastrinae

**Figure 5:**
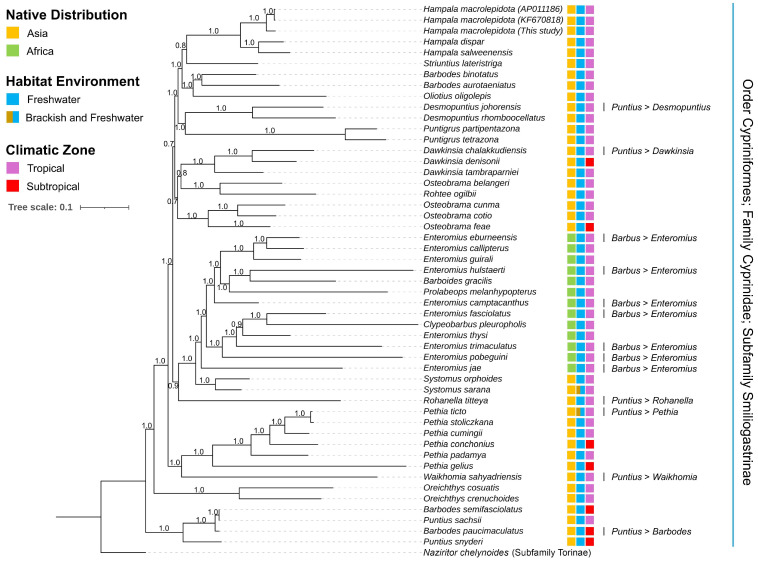
The Bayesian phylogenetic tree based on the 13 concatenated PCGs, describing the monophyletic evolutionary relationships of the three *Hampala* species within the Smiliogastrinae lineage, with high posterior probability supports for each node. The resulting cladogram supports a revised taxonomic classification and provides insights into the cladistic pattern of various species members within subfamily Smiliogastrinae. The native distribution range, environmental habitat, and climatic zone of each species are marked by different color boxes representing their distinctive clade.

Both BA and ML phylogenetic trees revealed a monophyletic clustering of subfamily Smiliogastrinae members, supported by high posterior probabilities and bootstrap values (Figure 5 and Figure S2). The phylogenetic trees classified genus *Hampala* as a monophyletic group, with the mitogenome of *H. macrolepidota* clustered together with its other congeneric species. The topologies also showed that *H. dispar* and *H. salweenensis*, which are distributed in Southeast Asian riverine systems, shared closer evolutionary relationships as sister species. Moreover, the mitogenomic topologies placed African cyprinids under the genera *Enteromius*, *Barboides*, *Prolabeops*, and *Clypeobarbus* as one group with a close relationship with the genus *Systoma*, which is distributed widely across Asia. The cladistic pattern also described the placement of monotypic *Rohanella titteya*, *Rohtee ogilbii*, and *Oliotius oligolepis* among other Smiliogastrinae species. The phylogenetic tree confirmed the taxonomic placement of previously ambiguous species in the Smiliogastrinae subfamily that are subject to revision, including six species formerly classified under the genus *Puntius* and seven species formerly under the genus *Barbus*. Notably, this revision presents monophyletic clusters in several genera such as *Dawkinsia*, *Desmopuntius*, *Systomus*, *Oreichthys*, *Puntigrus*, and *Pethia.* Moreover, non-monophyletic matrilineal lineage was observed in other genera, such as *Barbodes*, *Enteromius*, *Osteobrama*, and *Puntius*, which is indicated by the separate lineage of their species members, requiring further investigation. The topology analysis highlights the need for further mitogenome generation within the genera *Strintius*, *Waikhomia*, *Clypeobarbus*, and *Prolabeops* to clarify their classification among other congeners under the Smiliogastrinae lineage (Figure 5 and Figure S2).

Additionally, analysis based on partial *COI* sequences of *H. macrolepidota* revealed that one specimen from Vietnam was exclusively separated from the major clade, which generally comprised sequences from the Sundaic region (Indonesia, Malaysia, and Singapore). Nevertheless, within this Sundaic clade, sequences from China and from unknown localities were clustered together (Figure S3). In contrast, analysis of the partial *Cytb* gene detected three clades. Specifically, one clade was primarily composed of the Indonesian sequence from this study clustered with those from Malaysia, whereas another clade comprised sequences exclusively from Malaysia. The remaining clade included sequences from mainland Southeast Asia (Laos, Vietnam, and Thailand) together with sequences from China and from unknown localities (Figure S4).

### 3.7. Genetic Distance and Haplotype Diversity

**Figure 6:**
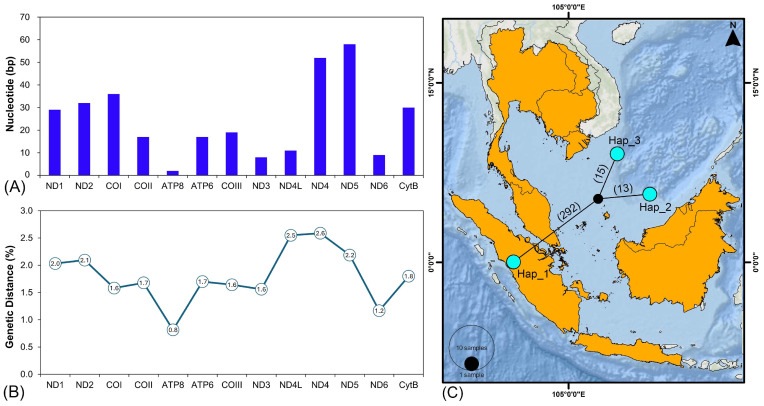
(**A**) Comparative analysis of K2P genetic distances across 13 PCGs of *H. macrolepidota*, highlighting inter-population variation across Southeast Asia. (**B**) Variation in the number of variable sites across the 13 PCGs in three sequences of *H. macrolepidota*. (**C**) The schematic TCS network illustrates the haplotype connectivity and distribution among the three mitogenomes of *H. macrolepidota* from both island (Sumatra, Indonesia) and likely mainland localities in Southeast Asia. The circle sizes reflect the haplotype frequency, while the mutation steps between haplotypes is shown by number in parentheses. The black circle denotes a median vector corresponding to a hypothetical haplotype.

The K2P genetic distance analysis utilizing the mitogenomes of the three *Hampala* species revealed notable intra-and interspecific variation at the species level. The mean value of interspecific genetic distance among *Hampala* species was 7.3%, with *H. dispar* and *H. salweenensis* indicating the lowest distance (7.6%), and the highest between *H. macrolepidota* and *H. salweenensis* (10.3%). Intraspecific variation within *H. macrolepidota* was estimated ranging from 0.25% to 2.8% (Table S9). Analysis of variable sites within each gene showed that *ND5* exhibited the highest variability, whereas *ATP8* contributed the least variation (Figure 6A). An in-depth analysis of each mitogenomic PCG showed that *ND4* exhibited the highest level of variation, whereas *ATP8* contributed the lowest (Figure 6B). Furthermore, inter-population genetic distance analyses revealed that the Indonesian population of *H. macrolepidota* exhibited low-to-moderate divergence from the mainland populations, ranging from 0 to 4.2% for the *COI* gene and 0 to 4.5% for the *Cytb* gene (Table S10). The haplotype analysis of *H. macrolepidota* based on complete mitogenome sequences identified three haplotypes, with 320 segregating sites, Hd = 1.0, and π = 0.6667. Among them, the Indonesian haplotype exhibited the highest divergence, separated by up to 292 mutational steps and forming a distinct haplotype compared to likely mainland populations, which differed by 15 and 13 mutational steps, respectively, from the median vectors (Figure 6C). In contrast, the haplotype analyses of the *COI* and *Cytb* genes identified 10 and 26 haplotypes, respectively, with 22 and 100 segregating sites. Both genes exhibited Hd = 1.00 and π = 0.29 for *COI* and 0.20 for *Cytb* (Figures S5 and S6; Tables S10 and S11). Notably, the *COI*-based haplotype network revealed three haplotypes shared across multiple geographic regions in Southeast and East Asia. Specifically, Hap_3 was detected in Indonesia, Malaysia, and Singapore; Hap_4 was detected in Indonesia and Malaysia; and Hap_10 was observed in China and in samples of unknown locality. The remaining seven haplotypes were unique and were confined to either mainland Southeast Asia or the Sundaic region (Figure S5; Table S11). Similarly, the *Cytb*-based haplotype network showed that only one shared haplotype (Hap_5) was distributed across Thailand, Laos, China, and samples of unknown locality. Conversely, the remaining 25 haplotypes were unique and obtained from either mainland or island ecosystems in Southeast Asia, as well as one sequence from unknown locality (Figure S6; Table S12).

## 4. Discussion

### 4.1. Mitogenome Characteristics

Prior to this study, the mitogenomes of *H. macrolepidota* were reported with only limited genetic characterization. Thus, the current study provides more detailed analysis of the newly characterized mitogenome of *H. macrolepidota* from its native range within the Sundaic Island ecosystem. Although varied in size, all mitogenome sequences of *H. macrolepidota* in this study exhibited significant similarity in terms of structural characterization and alignment with the typical mitochondrial architecture observed in other teleost fishes [2,3]. A comparative analysis of the mitogenome of *H. macrolepidota* with other species within genus *Hampala* revealed a similarity in nucleotide composition, with bias toward A+T and hydrophobic traits of the mitochondrial-encoded proteins [60]. The intergenic spacers and overlapping regions between mitochondrial genes in the *Hampala* species demonstrated a highly conserved structural organization, with minor variations observed in *tRNA-Leu1*, *tRNA-Leu2*, *tRNA-Asn*, *tRNA-Cys*, *tRNA-Ser1*, *tRNA-Ser2*, *tRNA-Asp*, and *ATP6*, indicating limited divergence in their mitogenomic architecture. Notably, the conserved overlap between *ATP8* and *ATP6*, along with length variations in the *ND5* and *ND6* genes among *Hampala* species, may reflect functional adaptations influencing the regulation of expression of mitochondrial genes in these fishes [5,61]. Furthermore, a consistent pattern of start and stop codons in the PCGs of *H. macrolepidota* and other congeneric species within the subfamily Smiliogastrinae was observed in this study, with ATG serving as the predominant start codon, whereas *COI* uniquely utilized GTG. A unique case was observed in *H. salweenensis*, which has an ATT type of start codon in its *ND3* gene in contrast with other species in the subfamily Smiliogastrinae, highlighting the need for further investigation to confirm gene structure and coding-sequence annotation. The use of ATG as a start codon is typical in teleost and other eukaryotic organisms, while alternative start codons remain comparatively uncommon [62]. This start and stop codon pattern also plays a significant role in verifying the PCGs through an open reading frame method, thereby preserving mitochondrial functionality and influencing evolutionary pathways [5]. In terms of the stop codon, we observed that *H. macrolepidota* and other congeneric species utilize both complete and incomplete stop codons (TA- and T--), which can be converted into full stop codons (TAA or AGA) through post-transcriptional polyadenylation during the RNA maturation process [9,63]. Such variations in termination codon usage are well documented among vertebrate mitogenomes and across diverse groups of teleost lineages [7].

The Ka/Ks ratio serves as a well-acknowledged metric for quantifying selective pressure in accordance with Darwinian evolutionary principles, enabling assessment of selection pressure at a molecular level across conspecific and phylogenetically proximate taxa [64]. Such a metric is essential for elucidating the evolutionary trajectories of PCGs in *H. macrolepidota* and offers valuable insights into species divergence driven by underlying molecular mechanisms [65]. Hence, this study indicated that pairwise Ka/Ks ratios between *H. macrolepidota* and its congeneric species within the subfamily Smiliogastrinae were all below ‘1’. This result indicates strong purifying selection with nonsynonymous mutations that are largely eliminated through synonymous substitutions, thereby reducing the potential negative impact on protein function [66]. Notably, among the 13 PCGs in *H. macrolepidota*, the *COI* gene exhibited the lowest Ka/Ks value, reflecting particularly strong purifying selection and highly constrained evolutionary rates. These findings further underscore the predominant role of purifying selection in suppressing deleterious mutations, a pattern consistent with the evolutionary conservation observed in other teleost mitogenomes [67]. The strong purifying selection operating on PCGs also confers evolutionary robustness to teleost fishes, maintaining genetic stability and enabling speciation through ecological adaptation and colonization of new ecosystems [68].

The PCGs among *Hampala* species showed a predominance of leucine, a hydrophobic amino acid with six codons, followed by three neutral amino acids (threonine, serine, and proline). This study also demonstrated that hydrophobic amino acids appear more frequently than hydrophilic ones, a pattern conserved across all *Hampala* species and broadly observed in teleost fishes [68]. The hydropathic properties of amino acids play a critical role in shaping the evolutionary trajectory of mitochondrial proteins, which are essential for metabolic regulation and environmental adaptation [69]. The findings of this study indicate that PCGs in *Hampala* are highly conserved and likely retain similar functional roles. Consequently, understanding these structural adaptations provides insights into how protein conformation and function are optimized in response to environmental stressors, highlighting their significance in evolutionary processes [70]. Moreover, the observed differences in RSCU values among *Hampala* species may correlate with gene expression levels, as highly expressed genes preferentially utilize optimal codons to enhance translational efficiency [71]. In addition, the newly sequenced mitogenome of *H. macrolepidota* encompassed both the small (*12S*) and large (*16S*) subunit rRNA genes encoded on the heavy strand, which is consistent with the arragement in other cyprinid species [9]. As essential components of ribonucleoprotein complexes, the rRNA genes of *H. macrolepidota* mediate the translation of genetic information from mRNA into proteins through precise molecular interactions [67]. Notably, our structural analysis indicated that most tRNAs in the *H. macrolepidota* mitogenome adopt a cloverleaf secondary structure, except for *tRNA-Ser1*. While this cloverleaf architecture is conserved across teleost mitogenomes, the frequent absence of the DHU arm in certain tRNAs represents a distinctive genomic feature [5]. However, the precise organization of tRNA genes in *H. macrolepidota*, coupled with the presence of duplications (heteroplasmy) in the WANCY region, plays a crucial role in both the expression and functional stability of mitochondrial genes [7,72]. Together, comprehending the genetic characteristics of both rRNA and tRNA elucidates fundamental aspects of mitochondrial genetic regulation of *H. macrolepidota*.

In addition, the comprehensive analysis of CRs in *Hampala* species reveals a conserved feature regarding the adenine and thymine preference, which is consistent with other teleost lineages [7]. The presence of four conserved sequence blocks (CSB-D, CSB-1, CSB-2, and CSB-3) in the CR for all *Hampala* species (except *H. dispar*) was also aligned with those previously observed in other fish mitogenomes [60,73]. The investigation of these conserved domains is significant due to the presence of highly conserved nucleotides within the highly variable CR. Therefore, the nucleotide variations detected within these conserved domains serve as critical markers that can be employed for examining population-level differentiation [5]. Moreover, this study further identified copies of tandem repeats within the ETAS region among *Hampala* species, revealing significant interspecific variation in repetitive motifs. This CR segment, rich in repetitive and highly variable sequences, contains motifs capable of forming stable hairpin loops that act as recognition signals for mitochondrial DNA replication termination [73].

### 4.2. Matrilineal Evolutionary Relationships

The integration of complete mitogenomic data is essential for reconstructing phylogenetic relationships and resolving taxonomic uncertainties arising from morphological plasticity and the high species richness of cyprinids [6,8,74,75]. Given the wide geographic range of this family, which has facilitated diversification across multiple biogeographic regions, comprehensive mitogenomic analyses are indispensable for elucidating their evolutionary paths and ecological adaptations. In this study, we established a robust phylogenetic framework for the subfamily Smiliogastrinae through multi-gene mitochondrial analyses, overcoming the limitations inherent to conventional single-locus approaches. Our mitogenome-based phylogeny recovered a well-supported monophyletic *Hampala* clade, consistent with previous studies. However, the current topology places them within the Smiliogastrinae, in contrast to earlier classifications that assigned the genus *Hampala* to Cyprininae or Barbinae based on only partial *Cytb* sequences, as well as *Cytb*, *16S rRNA*, and *D-loop* markers, respectively [25,30]. Moreover, the matrilineal phylogeny of the *Hampala* species also revealed a closer evolutionary affinity to *Striuntius*, in contrast to earlier mito-nuclear analyses that placed the genus nearer to *Puntigrus* [76]. This discrepancy underscores the value of enhanced phylogenetic resolution afforded by the expanded mitochondrial dataset used in this study. The recovered phylogenetic trees also corroborate earlier evidence of two distinct Asian cyprinid lineages separated by an intervening African clade [76], while reinforcing its value for further investigations into transcontinental diversification patterns. Additionally, our results further support the recent taxonomic revisions involving barbs historically placed within the genera *Puntius* and *Barbus* [11,13]. Notably, the non-monophyletic clustering of the South and Southeast Asian genus *Osteobrama* within the Smiliogastrinae lineage aligns with earlier findings derived from concatenated *Cytb* + *RAG1* datasets [77]. Similarly, the genus *Barbodes* also exhibited a non-monophyletic pattern, consistent with previous analyses using partial mitochondrial DNA and nuclear markers (*COI*, *Cytb*, and *Rp1*) [78]. Notably, the previous *Cytb*-based analysis indicates that *H. macrolepidota* comprises two major lineages, viz., an Indonesian clade and a mainland Southeast Asia–China clade [79]. However, the present phylogenetic inference based on both *COI* and *Cytb* genes revealed a mixed clustering pattern, in which the Indonesian island populations were coheshively clustered with mainland populations originating from multiple countries.

### 4.3. Genetic Distance, Haplotype Diversity, and Biogeographic Interpretation

The analysis of genetic distance and segregating sites across the 13 PCGs of *H. macrolepidota* revealed substantial variation in genetic divergence and polymorphism among genes, reflecting many differential selective pressures and molecular evolutionary rates. Notably, the *ND4* and *ND5* genes exhibited high levels of genetic variation, suggesting their potential utility as markers for assessing phylogeographic patterns within native *H. macrolepidota* populations in Southeast Asia. Additionally, elevated genetic distances and segregating sites were also observed in *COI* and *Cytb* genes, indicating that these loci are also suitable for population-level differentiation within the genus *Hampala*, consistent with previous studies [25,28,80]. Furthermore, the mitohaplotype network analysis identified three haplotypes of *H. macrolepidota*, including one from Indonesia and others from putative mainland Southeast Asian populations. However, the partial gene (*COI* and *Cytb*) analysis of *H. macrolepidota* in the present study revealed extensive haplotype clustering across Southeast Asia, reflecting substantial population genetic structure within the Sundaland biogeographic region. This pattern is likely influenced by the complex interplay of paleo-drainage connectivity, historical hydrological events, and geographic isolation [81,82,83]. Nonetheless, the presence of shared haplotypes between sequences from China and samples of unknown origin in the *COI* dataset may indicate human-mediated introductions into East Asia, given that the natural distribution of *H. macrolepidota* is largely restricted to island and mainland Southeast Asia. This interpretation aligns with earlier reports attributing the occurrence of the species in Hong Kong to the aquarium industry and aquaculture operations [16,17,18]. Consequently, this inference warrants further verification, as *Cytb* sequences from China clustered with those from Thailand and Laos. Thus, an alternative hypothesis suggests that the observed genetic affinity among *H. macrolepidota* populations from China, Thailand, and Laos reflects historical connectivity of river systems across Sundaland, potentially mediated by the paleo-Mekong River. This assumption is also supported by prior studies reporting the occurrence of *H. macrolepidota* in the lower reaches of the Lancang River in China, which represents the upper Mekong Basin and extends through southern Yunnan Province into Laos, Thailand, Cambodia, and Vietnam [17,36,82]. Nevertheless, robust evaluation of this hypothesis will require broader geographic sampling across the native range of *H. macrolepidota* in Southeast Asia and its putative distribution in southern China, particularly to mitigate potential biases arising from limited sample sizes. In contrast, the population connectivity of *H. macrolepidota* in the Sundaic region, including Peninsular Malaysia and Indonesian islands (Sumatra, Java, and Borneo) was likely shaped by multiple ancient river systems (Malacca Strait, North Sunda, and East Sunda), consistent with previous hypotheses regarding the diversification of other freshwater fishes [9,83,84,85]. Although currently separated by the sea, the Pleistocene low sea levels linked Southeast Asian landmasses, facilitating gene flow through freshwater networks and promoting subsequent divergence via geographic isolation [83,86]. Therefore, the present findings suggest that the distribution of shared and unique haplotypes of *H. macrolepidota* may reflect long-term population isolation in fragmented freshwater habitats, ultimately facilitating the emergence of independent population structures and local adaptations [86,87]. Based on our findings, we recommend that future studies on *H. macrolepidota* adopt high-resolution genomic approaches. These methods will enable a more comprehensive assessment of genetic diversity, population structure, and demographic dynamics of this cyprinid species across the Indo-Burma and Sundaland biodiversity hotspots.

## 5. Conclusions

This study provides a comprehensive mitogenomic characterization of *H. macrolepidota*, revealing structural variations that illuminate mechanisms of genus-level structural variations. The mitogenome-based phylogenetic inference and haplotype network analyses elucidate the matrilineal relationships among *Hampala* species and robustly resolve their systematic placement within the Smiliogastrinae lineage, while also revealing patterns of population genetic structure across both mainland and island populations. The partial analyses of *COI* and *Cytb* genes further reveal the population genetic structure of *H. macrolepidota* across Southeast and East Asia. The observed genetic divergence, coupled with well-supported phylogenetic clustering and the coexistence of shared and unique haplotypes among Indonesian populations, provide compelling evidence for long-term population isolation and localized adaptive differentiation. These genetic patterns are most plausibly shaped by ancient paleo-drainage connectivity and enduring geographic barriers that have structured population divergence over time. These findings highlight the need for mitogenomic data from the remaining seven *Hampala* species and the use of restriction-site-associated DNA sequencing, whole-genome sequencing, and single nucleotide polymorphism analyses to resolve the actual genetic diversity and population dynamics. Collectively, this study establishes a robust genomic baseline for future research in systematics, conservation genetics, and sustainable fishery management and conservation. Furthermore, it underscores the broader importance of expanding mitogenomic characterization across cyprinid taxa to enhance our understanding of evolutionary processes in freshwater ecosystems.

## Figures and Tables

**Table 1:** List of annotated genes, including their boundaries, sizes, and intergenic nucleotides (INs) for the newly sequenced *H. macrolepidota* mitogenome. ‘H’ and ‘L’ indicate the localization of genes on the heavy and light strands, respectively. The ‘-’ sign indicates an incomplete stop codon.

Gene	Start	Stop	Size (bp)	Strand	IN	Start Codon	Stop Codon	Anticodon
*tRNA-Phe* (*F*)	1	69	69	H	0			GAA
*12S rRNA*	70	1024	955	H	0			
*tRNA-Val* (*V*)	1025	1096	72	H	0			TAC
*16S rRNA*	1097	2770	1674	H	0			
*tRNA-Leu* (*L2*)	2771	2846	76	H	1			TAA
*ND1*	2848	3822	975	H	5	ATG	TAA	
*tRNA-Ile* (*I*)	3828	3899	72	H	−2			GAT
*tRNA-Gln* (*Q*)	3898	3968	71	L	1			TTG
*tRNA-Met* (*M*)	3970	4038	69	H	0			CAT
*ND2*	4039	5083	1045	H	0	ATG	T--	
*tRNA-Trp* (*W*)	5084	5154	71	H	0			TCA
*tRNA-Ala* (*A*)	5155	5223	69	L	1			TGC
*tRNA-Asn* (*N*)	5225	5297	73	L	35			GTT
*tRNA-Cys* (*C*)	5333	5399	67	L	0			GCA
*tRNA-Tyr* (*Y*)	5400	5466	67	L	1			GTA
*COI*	5468	7018	1551	H	0	GTG	TAA	
*tRNA-Ser* (*S2*)	7019	7089	71	L	1			TGA
*tRNA-Asp* (*D*)	7091	7162	72	H	8			GTC
*COII*	7171	7861	691	H	0	ATG	T--	
*tRNA-Lys* (*K*)	7862	7937	76	H	1			TTT
*ATP8*	7939	8103	165	H	−7	ATG	TAG	
*ATP6*	8097	8779	683	H	0	ATG	TA-	
*COIII*	8780	9564	785	H	0	ATG	TA-	
*tRNA-Gly* (*G*)	9565	9637	73	H	0			TCC
*ND3*	9638	9986	349	H	0	ATG	T--	
*tRNA-Arg* (*R*)	9987	10,056	70	H	0			TCG
*ND4L*	10,057	10,353	297	H	−7	ATG	TAA	
*ND4*	10,347	11,727	1381	H	0	ATG	T--	
*tRNA-His* (*H*)	11,728	11,796	69	H	0			GTG
*tRNA-Ser* (*S1*)	11,797	11,864	68	H	1			GCT
*tRNA-Leu* (*L1*)	11,866	11,939	74	H	3			TAG
*ND5*	11,943	13,766	1824	H	−4	ATG	TAA	
*ND6*	13,763	14,284	522	L	1	ATG	TAG	
*tRNA-Glu* (*E*)	14,286	14,354	69	L	5			TTC
*Cytb*	14,360	15,496	1137	H	4	ATG	TAA	
*tRNA-Thr* (*T*)	15,501	15,572	72	H	−2			TGT
*tRNA-Pro* (*P*)	15,571	15,641	71	L	0			TGG
Control region	15,642	17,104	1463					

**Table 2:** The nucleotide composition of *H. macrolepidota* mitogenomes (PP937078) and those of two congeners.

Species Name	Size (bp)	A%	T%	G%	C%	A + T%	G + C%	AT Skew	GC Skew
Complete Mitogenomes									
*H. macrolepidota* (PP937078)	17,104	33.69	24.97	14.80	26.54	58.66	41.34	0.149	−0.284
*H. macrolepidota* (AP011186)	17,120	33.55	24.94	14.92	26.59	58.49	41.51	0.147	−0.281
*H. macrolepidota* (KF670818)	16,765	33.51	24.71	14.94	26.84	58.22	41.78	0.151	−0.285
*H. dispar* (AP011245)	15,635	30.99	22.96	13.82	24.67	53.95	38.49	0.149	−0.282
*H. salweenensis* (MW548258)	16,913	33.62	25.34	14.70	26.34	58.96	41.04	0.140	−0.284
Protein-Coding Genes (PCGs)									
*H. macrolepidota* (PP937078)	11,405	32.76	25.42	13.50	28.32	58.18	41.82	0.126	−0.354
*H. macrolepidota* (AP011186)	11,405	31.23	26.97	14.47	27.33	58.20	41.80	0.073	−0.308
*H. macrolepidota* (KF670818)	11,407	31.26	26.99	14.47	27.28	58.25	41.75	0.073	−0.307
*H. dispar* (AP011245)	11,402	31.65	27.57	14.26	26.52	59.22	40.78	0.069	−0.301
*H. salweenensis* (MW548258)	11,401	31.30	27.54	14.33	26.83	58.84	41.16	0.064	−0.304
Ribosomal RNAs (rRNAs)									
*H. macrolepidota* (PP937078)	2629	35.72	19.09	20.16	25.03	54.81	45.19	0.303	−0.108
*H. macrolepidota* (AP011186)	2631	35.65	18.97	20.22	25.16	54.62	45.38	0.305	−0.109
*H. macrolepidota* (KF670818)	2631	35.69	18.89	20.22	25.20	54.58	45.42	0.308	−0.110
*H. dispar* (AP011245)	2631	35.84	19.99	20.03	24.14	55.83	44.17	0.284	−0.093
*H. salweenensis* (MW548258)	2627	35.82	19.83	19.98	24.36	55.65	44.35	0.287	−0.099
Transfer RNAs (tRNAs)									
*H. macrolepidota* (PP937078)	1561	31.64	24.25	18.77	25.34	55.89	44.11	0.132	−0.149
*H. macrolepidota* (AP011186)	1559	29.76	26.62	22.64	20.97	56.38	43.62	0.056	0.038
*H. macrolepidota* (KF670818)	1556	29.88	26.41	22.62	21.08	56.30	43.70	0.062	0.035
*H. dispar* (AP011245)	1557	29.74	26.78	22.74	20.75	56.52	43.48	0.052	0.046
*H. salweenensis* (MW548258)	1553	29.75	26.59	22.67	20.99	56.34	43.66	0.056	0.038
Control Regions (CRs)									
*H. macrolepidota* (PP937078)	1463	39.03	32.81	11.28	16.88	71.84	28.16	0.087	−0.199
*H. macrolepidota* (AP011186)	1477	37.78	32.23	12.73	17.26	70.01	29.99	0.079	−0.151
*H. macrolepidota* (KF670818)	1125	38.13	31.11	12.27	18.49	69.24	30.76	0.101	−0.202
*H. dispar* (AP011245)	-	-	-	-	-	-	-	-	-
*H. salweenensis* (MW548258)	1285	38.05	32.30	11.21	18.44	70.35	29.65	0.082	−0.244

## Data Availability

The data presented in this study can be found in GenBank (NCBI) at https://www.ncbi.nlm.nih.gov/ with the *COI* sequence accession number PX107884 and the complete mitogenome accession number PP937078.

## Supplementary Materials

The following supporting information can be downloaded at https://www.mdpi.com/article/10.3390/biom16020185/s1, Figure S1: The cloverleaf secondary structures of 22 tRNAs showing structural variation in *H. macroleidota*; Figure S2: The maximum-likelihood matrilineal phylogenetic tree constructed from the concatenated sequences of 13 PCGs illustrates the evolutionary relationships among cyprinids within the Smiliogastrinae subfamily; Figure S3: The Bayesian phylogenetic construction of *H. macrolepidota* based on partial *COI* gene sequences, including the newly reported mitogenome (marked with an asterisk) and 44 additional sequences retrieved from the GenBank database; Figure S4: The Bayesian phylogenetic construction of *H. macrolepidota* inferred from partial *Cytb* gene sequences derived from the reported mitogenome (marked with an asterisk) and 112 additional sequences obtained from the GenBank database; Figure S5: The TCS haplotype networks of *H. macrolepidota* across the Asian region based on partial *COI* gene sequences, generated using data from this study and sequences retrieved from the GenBank database; Figure S6: The TCS haplotype networks of *H. macrolepidota* across the Asian region based on partial *Cytb* gene sequences, generated using data from this study and sequences retrieved from the GenBank database; Table S1: Details of the mitogenomes of cyprinids within the Smiliogastrinae subfamily acquired from the GenBank database for phylogenetic analyses; Table S2: The partial mitochondrial *COI* and *Cytb* gene sequences of *H. macrolepidota* from this study and those obtained from the GenBank database; Table S3: The comparison of intergenic nucleotides of the *Hampala* species mitogenomes (five sequences); Table S4: The comparison of the start and stop codons of the PCGs across the *Hampala* species mitogenomes (five sequences); Table S5: The comparative analysis of pairwise Ka/Ks values for each PCG of *Hampala* species and other cyprinids within the Smiliogastrinae subfamily; Table S6: The abundance of amino acids and RSCU value of all 13 PCGs of three *Hampala* species (five dataset sequences); Table S7: The RSCU abundance and CDsPT calculation of the complete PCGs of *Hampala* mitogenomes; Table S8: Detailed comparison of anticodons found in the tRNA genes within the mitogenome of three *Hampala* species (five sequences); Table S9: The mean intra- and inter-species genetic distance among *Hampala* species based on the K2P model estimated from 13 concatenated PCG sequences; Table S10: The mean inter-population genetic distance within *H. macrolepidota* across Asian region based on the K2P model estimated from *COI* and *Cytb* partial gene sequences; Table S11: The haplotype composition of *H. macrolepidota* inferred from partial mitochondrial *COI* gene sequences obtained in this study and retrieved from the GenBank database; Table S12. The haplotype composition of *H. macrolepidota* inferred from partial mitochondrial *Cytb* gene sequences obtained in this study and retrieved from the GenBank database.
