# Supplementary material for: Mitogenomic Insights into the Hampala Barb (Hampala macrolepidota) from Sumatra, Indonesia: Characterization, Phylogenetic Placement, and Genetic Diversity
Source: Biomolecules. 2026 Jan 26;16(2):185. doi: 10.3390/biom16020185 (PMC12937876; doi:10.3390/biom16020185)
Supplement: Supplementary file 1 [file biomolecules-16-00185-s001.zip › biomolecules-4055776-supplementary.pdf]

Supplementary Materials

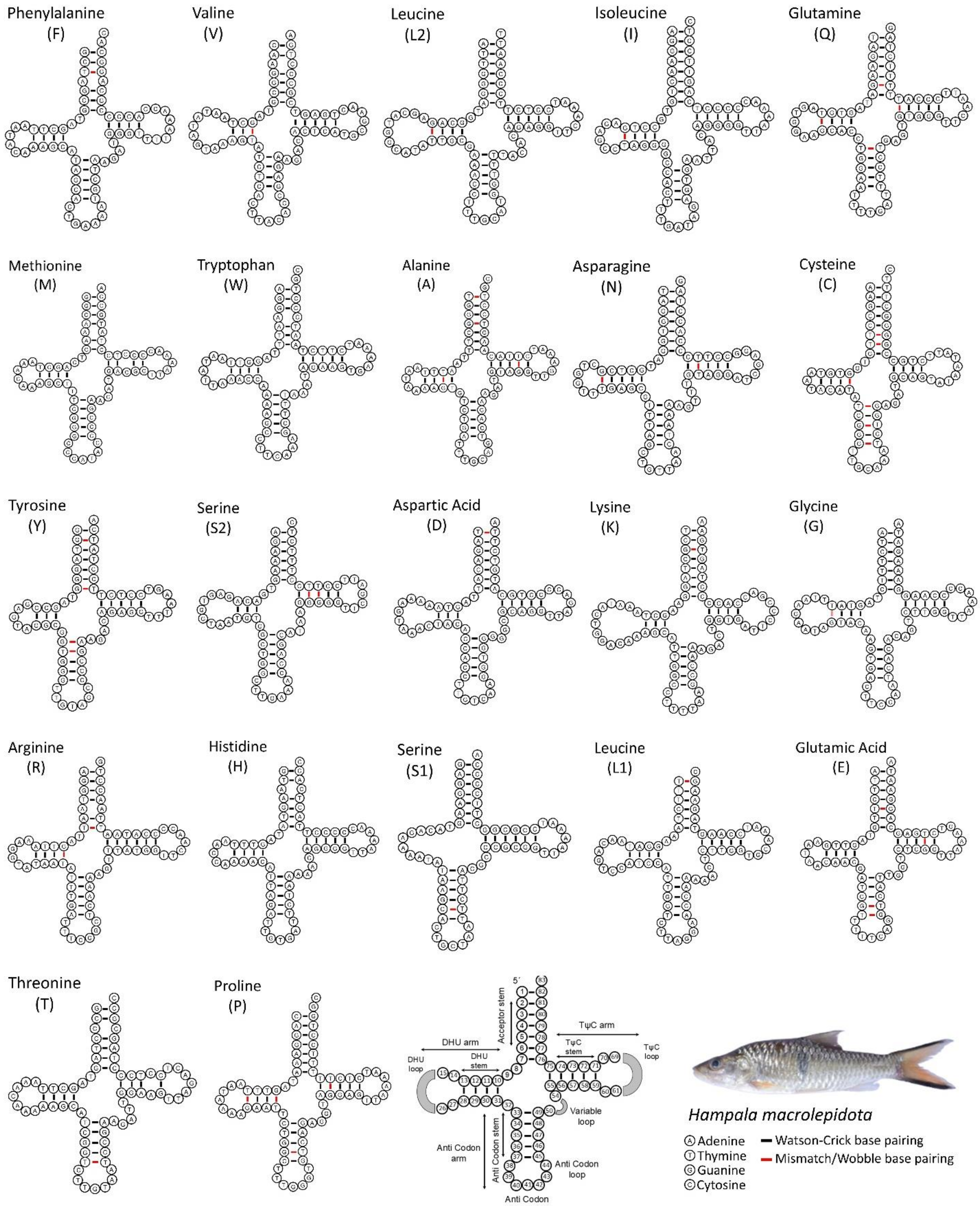

Figure S1. The cloverleaf secondary structures of 22 tRNAs showing structural variation in *H. macroleidota*. Each tRNA is labeled with its full name and corresponding IUPAC–IUB single-letter amino acid code. The Watson–Crick base pairs and mismatch/wobble base pairs are indicated by black and red bars, respectively.

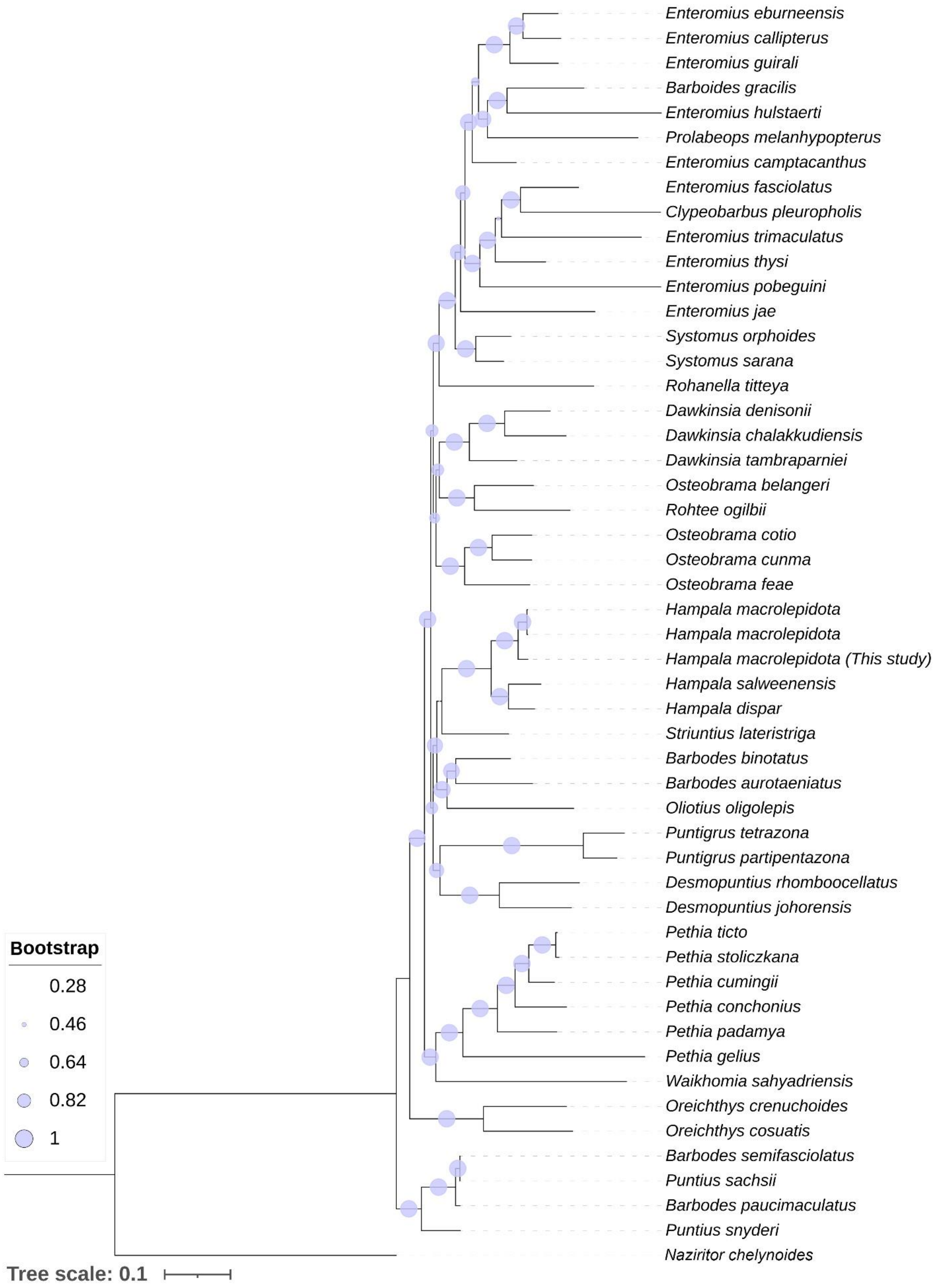

Figure S2. The maximum-likelihood matrilineal phylogenetic tree constructed from the concatenated sequences of 13 PCGs illustrates the evolutionary relationships among cyprinids within the Smiliogastrinae subfamily. The bootstrap support values are shown as purple circles at each node.

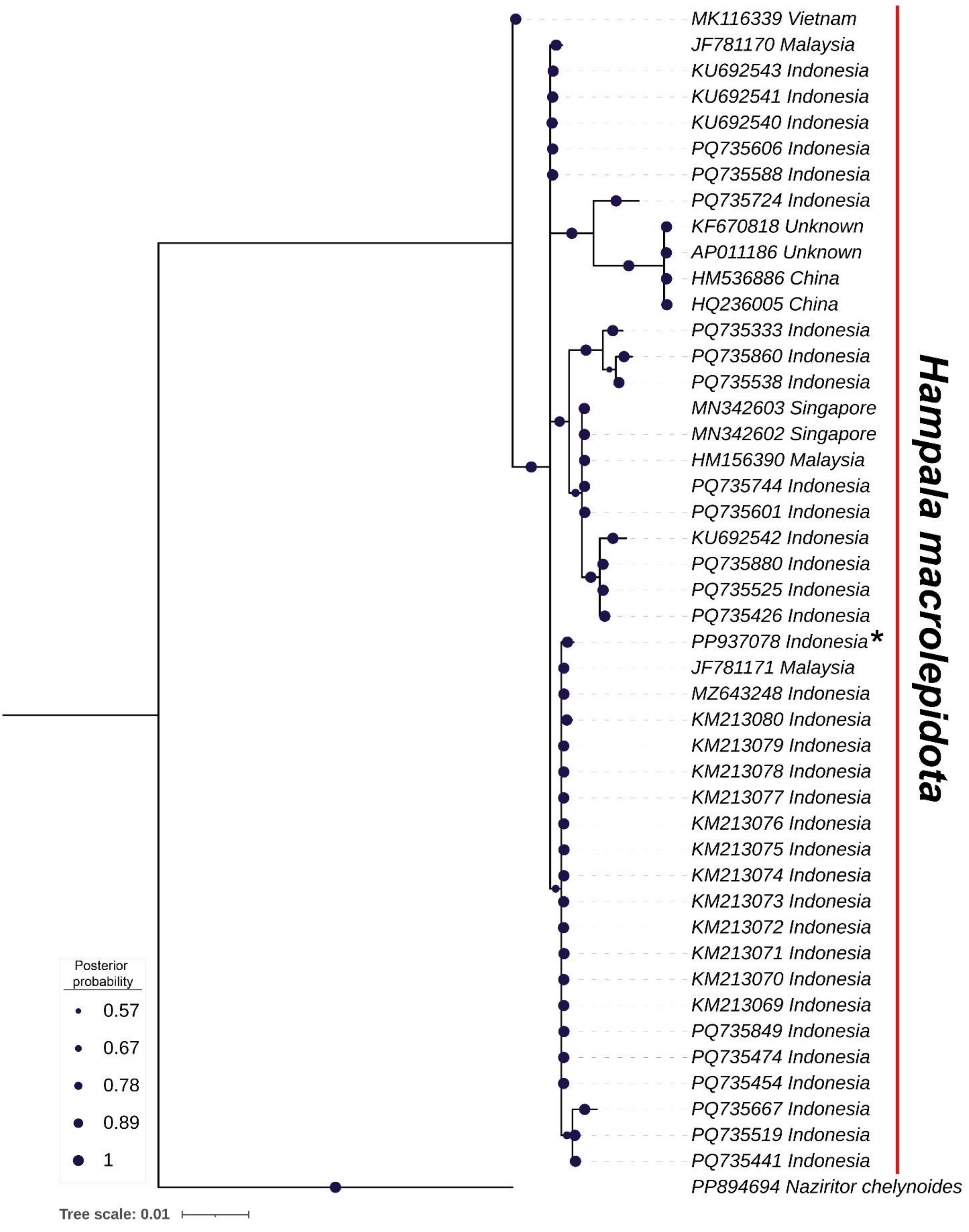

Figure S3. The Bayesian phylogenetic construction of *H. macrolepidota* based on partial *COI* gene sequences, including the newly reported mitogenome (marked with an asterisk) and 44 additional sequences retrieved from the GenBank database. The posterior probability values are indicated by blue circles at each node, representing the statistical support for inferred phylogenetic relationships.

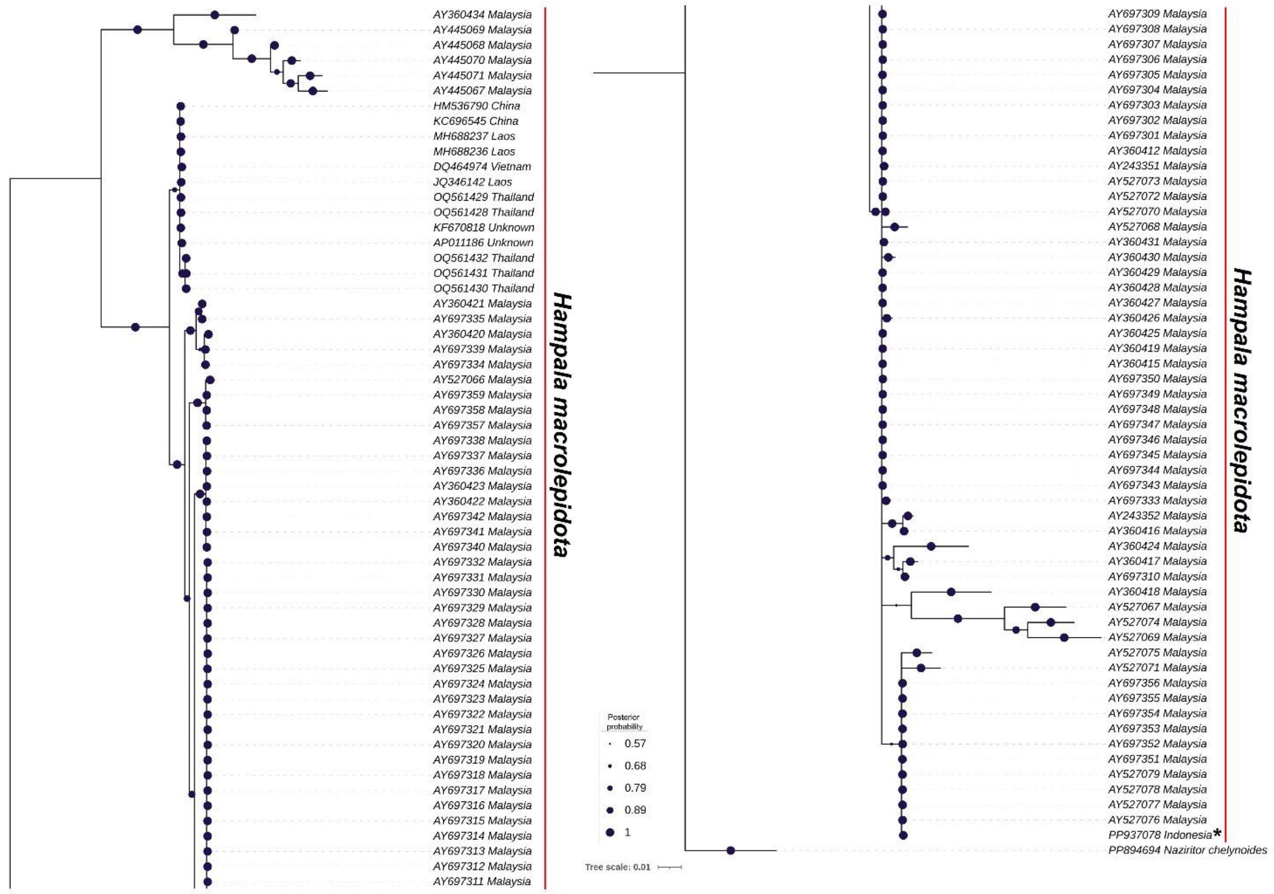

Figure S4. The Bayesian phylogenetic construction of *H. macrolepidota* inferred from partial *Cytb* gene sequences derived from the reported mitogenome (marked with an asterisk) and 112 additional sequences obtained from the GenBank database. The posterior probability values are shown as blue circles at each node, indicating the level of confidence in the constructed evolutionary relationships.

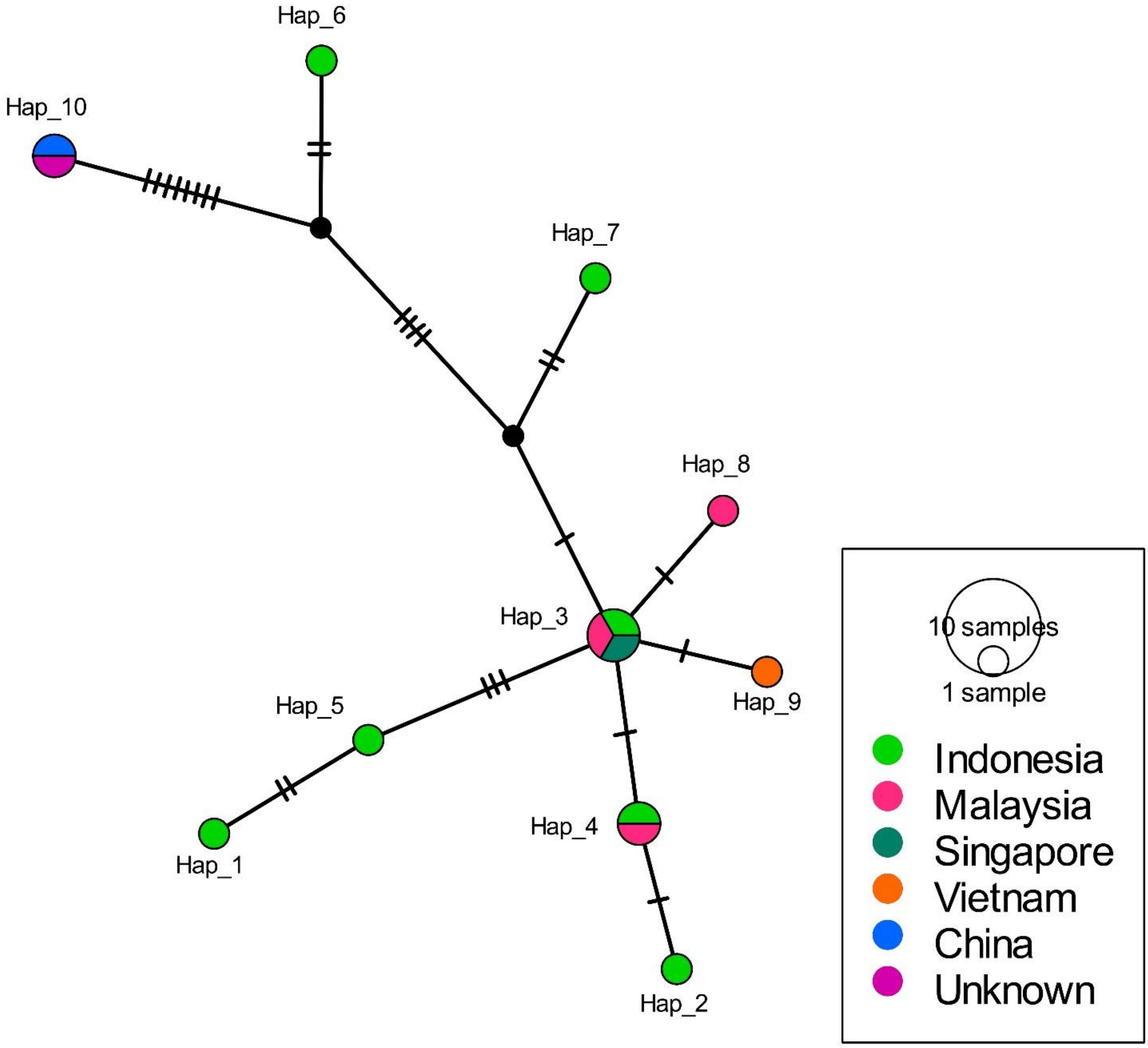

Figure S5. The TCS haplotype networks of *H. macrolepidota* across the Asian region based on partial *COI* gene sequences, generated using data from this study and sequences retrieved from the GenBank database. The circle sizes are proportional to haplotype frequencies, with different colors representing the localities. The numbers in parentheses indicate the number of mutational steps between haplotypes.

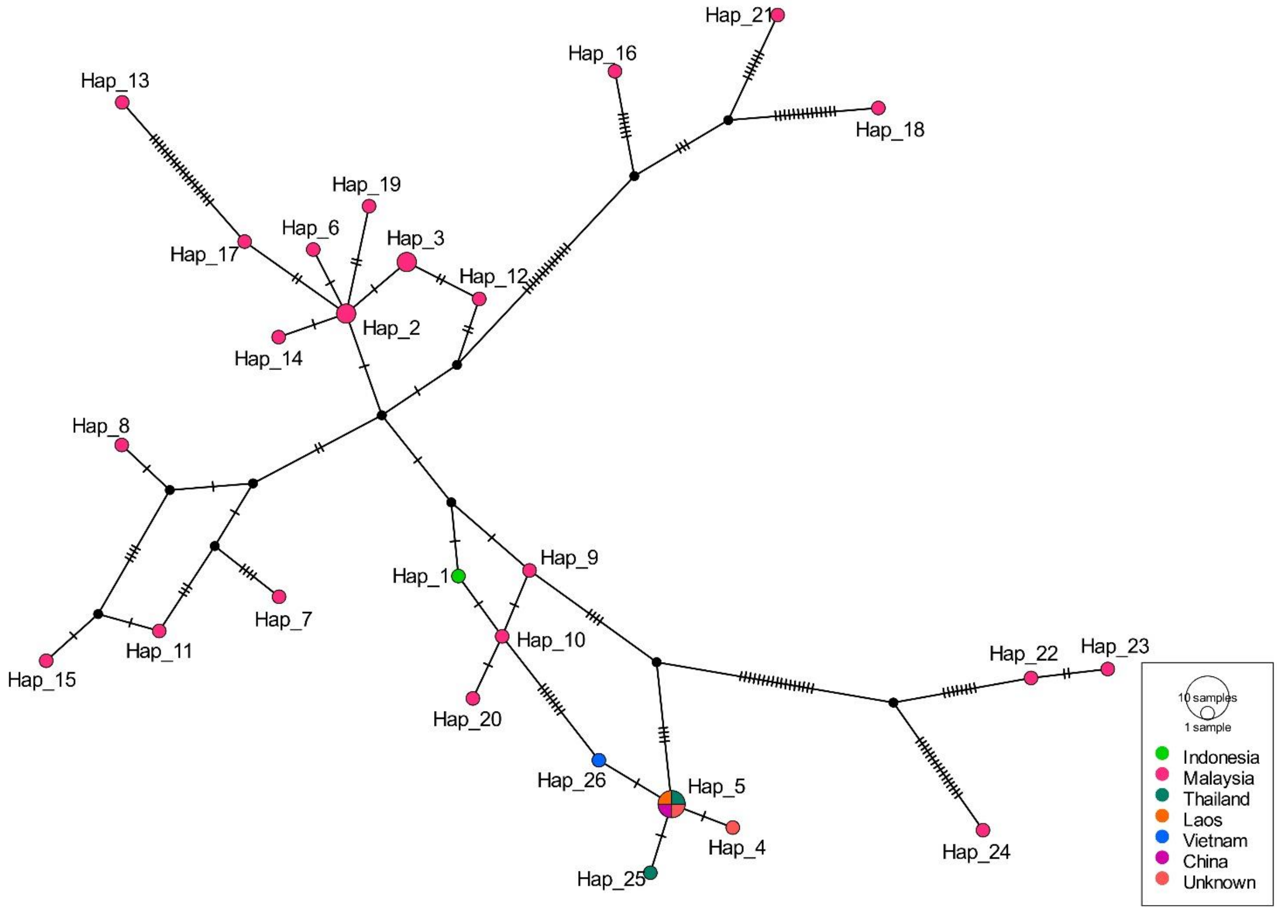

Figure S6. The TCS haplotype networks of *H. macrolepidota* across the Asian region based on partial *Cytb* gene sequences, generated using data from this study and sequences retrieved from the GenBank database. The circle sizes are proportional to haplotype frequencies, with different colors representing the localities. The numbers in parentheses indicate the number of mutational steps between haplotypes.

Table S1. Details of the mitogenomes of cyprinids within the Smiliogastrinae subfamily acquired from the GenBank database for phylogenetic analyses.

No	Species Name in GenBank	Species Valid Name	Accession Number	Reference
1	*Hampala macrolepidota*	*Hampala macrolepidota*	PP937078	This study
2	*Hampala macrolepidota*	*Hampala macrolepidota*	AP011186	Unpublished
3	*Hampala macrolepidota*	*Hampala macrolepidota*	KF670818	Liu et al. 2015
4	*Hampala dispar*	*Hampala dispar*	AP011245	Unpublished
5	*Hampala salweenensis*	*Hampala salweenensis*	MW548258	Unpublished
6	*Systomus orphoides*	*Systomus orphoides*	AP011189	Unpublished
7	*Systomus sarana*	*Systomus sarana*	KU886061	Biswal et al. 2017
8	*Puntius eugrammus*	*Desmopuntius johorensis*	AP011369	Unpublished
9	*Desmopuntius rhomboocellatus*	*Desmopuntius rhomboocellatus*	AP011309	Unpublished
10	*Puntius paucimaculatus*	*Barbodes paucimaculatus*	OR264466	Unpublished
11	*Puntius semifasciolatus*	*Barbodes semifasciolatus*	KC113209	Jang-Liaw et al. 2013
12	*Barbodes binotatus*	*Barbodes binotatus*	KY305681	Unpublished
13	*Barbodes aurotaeniatus*	*Barbodes aurotaeniatus*	AP011381	Unpublished
14	*Puntigrus partipentazona*	*Puntigrus partipentazona*	AP011368	Unpublished
15	*Puntius tetrazona*	*Puntigrus tetrazona*	EU287909	Unpublished
16	*Puntius ticto*	*Pethia ticto*	AB238969	Saitoh et al. 2006
17	*Pethia conchonius*	*Pethia conchonius*	PP059109	Xu et al. 2015
18	*Pethia stoliczkana*	*Pethia stoliczkana*	OP785085	Qiao et al. 2024
19	*Pethia padamya*	*Pethia padamya*	ON864408	Pan et al. 2023
20	*Pethia gelius*	*Pethia gelius*	AP013336	Unpublished
21	*Pethia cumingii*	*Pethia cumingii*	AP011316	Unpublished
22	*Osteobrama cunma*	*Osteobrama cunma*	AP011261	Unpublished
23	*Osteobrama feae*	*Osteobrama feae*	AP011262	Unpublished
24	*Osteobrama cotio*	*Osteobrama cotio*	AP011260	Unpublished
25	*Osteobrama belangeri*	*Osteobrama belangeri*	MK749691	Behera et al. 2019
26	*Oreichthys cosuatis*	*Oreichthys cosuatis*	AP011366	Unpublished
27	*Oreichthys crenuchoides*	*Oreichthys crenuchoides*	MK456608	Unpublished
28	*Enteromius pobeguini*	*Enteromius pobeguini*	AP012061	Unpublished
29	*Enteromius jae*	*Enteromius jae*	AP011407	Unpublished
30	*Enteromius camptacanthus*	*Enteromius camptacanthus*	AP011378	Unpublished
31	*Barbus trimaculatus*	*Enteromius trimaculatus*	AB239600	Saitoh et al. 2006
32	*Enteromius fasciolatus*	*Enteromius fasciolatus*	AP011377	Unpublished
33	*Enteromius eburneensis*	*Enteromius eburneensis*	AP011379	Unpublished
34	*Enteromius thysi*	*Enteromius thysi*	OP819561	Kundu et al. 2023
35	*Enteromius hulstaerti*	*Enteromius hulstaerti*	AP011194	Unpublished
36	*Enteromius guirali*	*Enteromius guirali*	AP009314	Unpublished
37	*Enteromius callipterus*	*Enteromius callipterus*	AP009313	Unpublished
38	*Puntius chalakkudiensis*	*Dawkinsia chalakkudiensis*	JX311437	Unpublished
39	*Dawkinsia denisonii*	*Dawkinsia denisonii*	AP011244	Unpublished
40	*Dawkinsia tambraparniei*	*Dawkinsia tambraparniei*	AP011374	Unpublished
41	*Clypeobarbus pleuropholis*	*Clypeobarbus pleuropholis*	AP011408	Unpublished
42	*Barboides gracilis*	*Barboides gracilis*	AP011241	Unpublished
43	*Oliotius oligolepis*	*Oliotius oligolepis*	ON864407	Sun et al. 2023
44	*Striuntius lateristriga*	*Striuntius lateristriga*	AP011318	Unpublished
45	*Puntius sahyadriensis*	*Waikhomia sahyadriensis*	AP012065	Unpublished
46	*Puntius titteya*	*Rohanella titteya*	PQ720779	Unpublished

47	*Puntius snyderi*	*Puntius snyderi*	KC113210	Jang-Liaw et al. 2013
48	*Puntius sachsii*	*Puntius sachsii*	MZ364158	Unpublished
49	*Prolabeops melanhypopterus*	*Prolabeops melanhypopterus*	ON323519	Schedel et al. 2022
50	*Rohtee ogilbii*	*Rohtee ogilbii*	AP011362	Unpublished

Table S2. The partial mitochondrial *COI* and *Cytb* gene sequences of *H. macrolepidota* from this study and those obtained from the GenBank database. '*n*' denotes the number of sequences analyzed.

No	Accession Number	Gene	*n*	Locality	Reference
1	PP937078	*COI*	1	Indonesia	This study
2	PQ735333, PQ735426, PQ735441, PQ735454, PQ735474, PQ735519, PQ735525, PQ735538, PQ735588, PQ735601, PQ735606, PQ735667, PQ735724, PQ735744, PQ735849, PQ735860, PQ735880	*COI*	17	Indonesia	Delrieu-Trottin et al. 2025
3	KM213069 – 213080	*COI*	12	Indonesia	Makmur et al. 2014
4	KU692540 – 692543	*COI*	4	Indonesia	Dahruddin et al. 2017
5	MZ643248	*COI*	1	Indonesia	Wulandari 2021
6	JF781170 – 781171	*COI*	2	Malaysia	Song et al. 2013
7	HM156390	*COI*	1	Malaysia	Song et al. 2013
8	MN342602 – 342603	*COI*	2	Singapore (aquarium trade)	Collins et al. 2012
9	MK116339	*COI*	1	Vietnam	Vu et al. 2018
10	HQ236005	*COI*	1	China	Wang et al. 2016
11	HM536886	*COI*	1	China	Yang et al. 2010
12	AP011186	*COI*	1	Unknown	Unpublished
13	KF670818	*COI*	1	Unknown	Liu et al. 2015
14	PP937078	*Cytb*	1	Indonesia	This study
15	AY697301 – 697359	*Cytb*	59	Malaysia	Ryan & Esa 2006
16	AY360415 – 360431	*Cytb*	17	Malaysia	Ryan & Esa 2006
17	AY527066 – 527079	*Cytb*	14	Malaysia	Ryan & Esa 2006
18	AY445067 – 445071	*Cytb*	5	Malaysia	Ryan & Esa 2006
19	AY243351 – 243352	*Cytb*	2	Malaysia	Esa et al. 2012
20	AY360412, AY360434	*Cytb*	2	Malaysia	Ryan & Esa 2006
21	OQ561428 – 561432	*Cytb*	5	Thailand	Panprommin et al. 2023
22	MH688236 – 688237	*Cytb*	2	Laos	Gillet et al. 2018
23	JQ346142	*Cytb*	1	Laos	Pasco-Viel et al. 2012
24	DQ464974	*Cytb*	1	Vietnam	Thai et al. 2007
25	KC696545	*Cytb*	1	China	Wang et al. 2013
26	HM536790	*Cytb*	1	China	Yang et al. 2010
27	AP011186	*Cytb*	1	Unknown	Unpublished
28	KF670818	*Cytb*	1	Unknown	Liu et al. 2015

Table S3. The comparison of intergenic nucleotides of the *Hampala* species mitogenomes (five sequences).

Genes	*H. macrolepidota* (PP937078)	*H. salweenensis* (MW548258)	*H. dispar* (AP011245)	*H. macrolepidota* (KF670818)	*H. macrolepidota* (AP011186)
tRNA-Phe (F)	0	0	0	0	0
12S rRNA	0	0	0	0	0
tRNA-Val (V)	0	0	0	0	0
16S rRNA	0	0	0	0	0
tRNA-Leu (L2)	1	1	1	2	1
ND1	5	5	5	5	5
tRNA-Ile (I)	-2	-2	-2	-2	-2
tRNA-Gln (Q)	1	1	1	1	1
tRNA-Met (M)	0	0	0	0	0
ND2	0	0	0	0	0
tRNA-Trp (W)	0	0	0	0	0
tRNA-Ala (A)	1	1	1	1	1
tRNA-Asn (N)	35	35	34	34	34
tRNA-Cys (C)	0	1	0	1	0
tRNA-Tyr (Y)	1	1	1	1	1
COI	0	0	0	0	0
tRNA-Ser (S2)	1	2	1	2	1
tRNA-Asp (D)	8	5	4	9	8
COII	0	0	0	0	0
tRNA-Lys (K)	1	1	1	1	1
ATP8	-7	-7	-7	-7	-7
ATP6	0	-1	0	-1	0
COIII	0	0	0	0	0
tRNA-Gly (G)	0	0	0	0	0
ND3	0	0	0	0	0
tRNA-Arg (R)	0	0	1	0	0
ND4L	-7	-7	-7	-7	-7
ND4	0	0	0	0	0
tRNA-His (H)	0	0	1	0	1
tRNA-Ser (S1)	1	0	3	0	3
tRNA-Leu (L1)	3	8	3	4	3
ND5	-4	-4	-4	-4	-4
ND6	1	0	1	0	1
tRNA-Glu (E)	5	5	5	5	5
Cytb	4	4	4	4	4
tRNA-Thr (T)	-2	-2	-2	-2	-2
tRNA-Pro (P)	0	0	0	0	0
Control region					

Table S4. The comparison of the start and stop codons of the PCGs across the *Hampala* species mitogenomes (five sequences).

PCGs	***H. macrolepidota* (PP937078)**		***H. salweenensis* (MW548258)**		***H. dispar* (AP011245)**		***H. macrolepidota* (KF670818)**		***H. macrolepidota* (AP011186)**	
	**Start**	**Stop**	**Start**	**Stop**	**Start**	**Stop**	**Start**	**Stop**	**Start**	**Stop**
ND1	ATG	TAA	ATG	TAA	ATG	TAA	ATG	TAA	ATG	TAA
ND2	ATG	T--	ATG	T--	ATG	T--	ATG	T--	ATG	T--
COI	GTG	TAA	GTG	TAA	GTG	TAA	GTG	TAA	GTG	TAA
COII	ATG	T--	ATG	T--	ATG	T--	ATG	T--	ATG	T--
ATP8	ATG	TAG	ATG	TAA	ATG	TAG	ATG	TAG	ATG	TAG
ATP6	ATG	TA-	ATG	TAA	ATG	TA-	ATG	TAA	ATG	TA-
COIII	ATG	TA-	ATG	TAA	ATG	TA-	ATG	TA-	ATG	TAA
ND3	ATG	T--	ATT	T--	ATG	T--	ATG	T--	ATG	T--
ND4L	ATG	TAA	ATG	TAA	ATG	TAA	ATG	TAA	ATG	TAA
ND4	ATG	T--	ATG	T--	ATG	T--	ATG	T--	ATG	T--
ND5	ATG	TAA	ATG	TAA	ATG	TAA	ATG	TAA	ATG	TAA
ND6	ATG	TAG	ATG	TAA	ATG	TAG	ATG	TAG	ATG	TAG
Cytb	ATG	TAA	ATG	TAA	ATG	TAA	ATG	TAA	ATG	TAA

Table S5. The comparative analysis of pairwise Ka/Ks values for each PCG of *Hampala* species and other cyprinids within the Smiliogastrinae subfamily.

**Species**	**Species**	***ND1***	***ND2***	***COI***	***COII***	***ATP8***	***ATP6***	***COIII***	***ND3***	***ND4L***	***ND4***	***ND5***	***ND6***	***Cytb***
Hampala macrolepidota	*Hampala macrolepidota*	0.028	0.101	0.000	0.000	0.000	0.045	0.142	0.068	0.000	0.024	0.042	0.000	0.022
Hampala macrolepidota	*Hampala macrolepidota*	0.028	0.091	0.000	0.000	0.278	0.023	0.116	0.056	0.000	0.027	0.042	0.043	0.012
Hampala macrolepidota	*Hampala macrolepidota*	0.042	0.043	0.000	0.011	0.143	0.031	0.016	0.036	0.000	0.025	0.041	0.051	0.026
Hampala macrolepidota	*Hampala dispar*	0.062	0.060	0.005	0.010	0.349	0.029	0.027	0.068	0.000	0.038	0.056	0.059	0.044
Hampala macrolepidota	*Hampala salweenensis*	0.042	0.089	0.023	0.051	0.150	0.032	0.029	0.030	0.010	0.040	0.066	0.104	0.051
Hampala macrolepidota	*Systomus orphoides*	0.051	0.092	0.017	0.062	0.105	0.023	0.024	0.028	0.015	0.051	0.050	0.125	0.040
Hampala macrolepidota	*Systomus sarana*	0.077	0.101	0.019	0.080	0.115	0.039	0.047	0.026	0.011	0.058	0.061	0.101	0.080
Hampala macrolepidota	*Desmopuntius johorensis*	0.061	0.112	0.018	0.103	0.117	0.047	0.042	0.024	0.017	0.059	0.064	0.118	0.053
Hampala macrolepidota	*Desmopuntius rhomboocellatus*	0.044	0.109	0.015	0.046	0.082	0.033	0.021	0.050	0.011	0.046	0.063	0.090	0.031
Hampala macrolepidota	*Barbodes paucimaculatus*	0.045	0.107	0.017	0.043	0.082	0.029	0.022	0.032	0.012	0.045	0.060	0.083	0.039
Hampala macrolepidota	*Barbodes semifasciolatus*	0.058	0.110	0.016	0.042	0.122	0.039	0.014	0.027	0.016	0.054	0.069	0.127	0.059
Hampala macrolepidota	*Barbodes binotatus*	0.072	0.180	0.015	0.044	0.180	0.047	0.014	0.029	0.015	0.051	0.060	0.073	0.048
Hampala macrolepidota	*Barbodes aurotaeniatus*	0.044	0.108	0.015	0.053	0.082	0.034	0.020	0.027	0.010	0.049	0.064	0.076	0.039
Hampala macrolepidota	*Puntigrus partipentazona*	0.150	0.233	0.018	0.077	0.186	0.071	0.034	0.091	0.077	0.078	0.141	0.204	0.059
Hampala macrolepidota	*Puntigrus tetrazona*	0.059	0.103	0.021	0.069	0.147	0.088	0.047	0.057	0.039	0.050	0.077	0.128	0.033
Hampala macrolepidota	*Pethia ticto*	0.047	0.105	0.025	0.070	0.106	0.073	0.038	0.046	0.025	0.044	0.068	0.154	0.026
Hampala macrolepidota	*Pethia conchonius*	0.046	0.105	0.025	0.070	0.106	0.073	0.037	0.046	0.025	0.044	0.068	0.154	0.026
Hampala macrolepidota	*Pethia stoliczkana*	0.062	0.099	0.021	0.068	0.164	0.091	0.047	0.065	0.041	0.048	0.076	0.125	0.035
Hampala macrolepidota	*Pethia padamya*	0.065	0.133	0.033	0.064	0.143	0.065	0.033	0.047	0.037	0.050	0.056	0.178	0.036
Hampala macrolepidota	*Pethia gelius*	0.100	0.234	0.026	0.093	0.615	0.119	0.051	0.066	0.074	0.090	0.101	0.370	0.110
Hampala macrolepidota	*Pethia cumingii*	0.060	0.130	0.024	0.070	0.184	0.073	0.028	0.040	0.032	0.058	0.072	0.134	0.041
Hampala macrolepidota	*Osteobrama cunma*	0.062	0.102	0.030	0.051	0.159	0.089	0.032	0.050	0.019	0.047	0.062	0.086	0.050
Hampala macrolepidota	*Osteobrama feae*	0.052	0.106	0.026	0.046	0.166	0.053	0.019	0.039	0.021	0.035	0.082	0.088	0.074
Hampala macrolepidota	*Osteobrama cotio*	0.053	0.110	0.033	0.063	0.137	0.060	0.027	0.037	0.030	0.058	0.067	0.102	0.057
Hampala macrolepidota	*Osteobrama belangeri*	0.052	0.118	0.010	0.042	0.127	0.039	0.043	0.023	0.005	0.046	0.078	0.132	0.045
Hampala macrolepidota	*Oreichthys cosuatis*	0.068	0.135	0.013	0.041	0.269	0.055	0.031	0.045	0.024	0.059	0.092	0.174	0.052
Hampala macrolepidota	*Oreichthys crenuchoides*	0.042	0.128	0.016	0.040	0.163	0.073	0.034	0.013	0.014	0.043	0.090	0.106	0.068
Hampala macrolepidota	*Enteromius pobeguini*	0.065	0.196	0.013	0.047	0.156	0.049	0.060	0.039	0.049	0.052	0.118	0.115	0.029
Hampala macrolepidota	*Enteromius jae*	0.099	0.117	0.024	0.072	0.163	0.068	0.056	0.062	0.039	0.061	0.072	0.167	0.069

Hampala macrolepidota	*Enteromius camptacanthus*	0.049	0.101	0.015	0.050	0.081	0.048	0.031	0.045	0.010	0.054	0.057	0.097	0.066
Hampala macrolepidota	*Enteromius trimaculatus*	0.064	0.096	0.027	0.063	0.305	0.089	0.065	0.058	0.032	0.048	0.090	0.173	0.053
Hampala macrolepidota	*Enteromius fasciolatus*	0.063	0.085	0.027	0.088	0.151	0.050	0.058	0.022	0.024	0.060	0.080	0.155	0.093
Hampala macrolepidota	*Enteromius eburneensis*	0.050	0.090	0.014	0.039	0.106	0.047	0.037	0.018	0.019	0.052	0.059	0.108	0.052
Hampala macrolepidota	*Enteromius thysi*	0.053	0.066	0.017	0.045	0.083	0.028	0.040	0.042	0.003	0.048	0.055	0.126	0.048
Hampala macrolepidota	*Enteromius hulstaerti*	0.046	0.140	0.033	0.108	0.250	0.095	0.057	0.018	0.043	0.094	0.079	0.145	0.084
Hampala macrolepidota	*Enteromius guirali*	0.051	0.098	0.013	0.036	0.088	0.039	0.029	0.021	0.011	0.044	0.067	0.115	0.044
Hampala macrolepidota	*Enteromius callipterus*	0.047	0.108	0.015	0.049	0.080	0.046	0.039	0.016	0.016	0.043	0.067	0.134	0.067
Hampala macrolepidota	*Dawkinsia chalakkudiensis*	0.108	0.158	0.019	0.049	0.144	0.042	0.026	0.121	0.066	0.075	0.080	0.233	0.066
Hampala macrolepidota	*Dawkinsia denisonii*	0.085	0.160	0.012	0.035	0.133	0.045	0.024	0.081	0.038	0.058	0.078	0.131	0.048
Hampala macrolepidota	*Dawkinsia tambraparniei*	0.068	0.109	0.014	0.044	0.095	0.046	0.023	0.043	0.015	0.053	0.082	0.100	0.051
Hampala macrolepidota	*Clypeobarbus pleuropholis*	0.112	0.179	0.042	0.171	0.342	0.174	0.058	0.035	0.046	0.067	0.085	0.144	0.146
Hampala macrolepidota	*Barboides gracilis*	0.036	0.083	0.017	0.034	0.117	0.040	0.044	0.052	0.022	0.054	0.044	0.093	0.044
Hampala macrolepidota	*Oliotius oligolepis*	0.083	0.123	0.019	0.049	0.145	0.069	0.027	0.031	0.047	0.055	0.097	0.159	0.060
Hampala macrolepidota	*Striuntius lateristriga*	0.058	0.087	0.011	0.032	0.095	0.029	0.025	0.048	0.016	0.054	0.055	0.112	0.044
Hampala macrolepidota	*Waikhomia sahyadriensis*	0.069	0.123	0.023	0.061	0.134	0.101	0.023	0.064	0.020	0.061	0.079	0.098	0.040
Hampala macrolepidota	*Rohanella titteya*	0.050	0.118	0.028	0.093	0.127	0.092	0.040	0.040	0.022	0.056	0.067	0.145	0.040
Hampala macrolepidota	*Puntius snyderi*	0.039	0.128	0.015	0.051	0.085	0.029	0.020	0.033	0.009	0.053	0.062	0.100	0.031
Hampala macrolepidota	*Puntius sachsii*	0.039	0.128	0.015	0.051	0.085	0.029	0.020	0.033	0.009	0.053	0.062	0.100	0.031
Hampala macrolepidota	*Prolabeops melanhypopterus*	0.042	0.099	0.019	0.060	0.186	0.063	0.058	0.048	0.016	0.057	0.063	0.087	0.045
Hampala macrolepidota	*Rohtee ogilbii*	0.060	0.106	0.011	0.034	0.183	0.063	0.059	0.026	0.025	0.055	0.081	0.179	0.039
Average		0.062	0.120	0.018	0.056	0.156	0.057	0.039	0.045	0.025	0.053	0.072	0.126	0.051
STDEV		0.026	0.042	0.008	0.028	0.094	0.028	0.023	0.022	0.020	0.014	0.019	0.055	0.023

Table S6. The abundance of amino acids and RSCU value of all 13 PCGs of three *Hampala* species (five dataset sequences). The amino acid showed as single-letter code in parenthesis, while stop codons showed as asterisk.

***Hampala macrolepidota* (PP937078)**											
Codon	Count	RSCU	Codon	Count	RSCU	Codon	Count	RSCU	Codon	Count	RSCU
UUU(F)	69	0.94	UCU(S)	50	0.92	UAU(Y)	91	1.24	UGU(C)	28	0.81
UUC(F)	78	1.06	UCC(S)	38	0.70	UAC(Y)	56	0.76	UGC(C)	41	1.19
UUA(L)	61	0.86	UCA(S)	59	1.09	UAA(*)	27	0.69	UGA(W)	69	1.27
UUG(L)	22	0.31	UCG(S)	5	0.09	UAG(*)	15	0.38	UGG(W)	40	0.73
CUU(L)	110	1.55	CCU(P)	117	1.50	CAU(H)	89	0.95	CGU(R)	22	0.61
CUC(L)	70	0.99	CCC(P)	68	0.87	CAC(H)	99	1.05	CGC(R)	53	1.46
CUA(L)	118	1.67	CCA(P)	102	1.31	CAA(Q)	82	1.71	CGA(R)	41	1.13
CUG(L)	44	0.62	CCG(P)	24	0.31	CAG(Q)	14	0.29	CGG(R)	29	0.80
AUU(I)	185	1.38	ACU(T)	134	1.48	AAU(N)	151	1.04	AGU(S)	61	1.12
AUC(I)	83	0.62	ACC(T)	84	0.93	AAC(N)	140	0.96	AGC(S)	113	2.08
AUA(M)	100	1.38	ACA(T)	117	1.29	AAA(K)	96	1.76	AGA(*)	42	1.07
AUG(M)	45	0.62	ACG(T)	27	0.30	AAG(K)	13	0.24	AGG(*)	73	1.86
GUU(V)	34	1.10	GCU(A)	30	0.86	GAU(D)	14	0.60	GGU(G)	26	0.72
GUC(V)	21	0.68	GCC(A)	57	1.63	GAC(D)	33	1.40	GGC(G)	33	0.92
GUA(V)	62	2.00	GCA(A)	48	1.37	GAA(E)	46	1.84	GGA(G)	58	1.61
GUG(V)	7	0.23	GCG(A)	5	0.14	GAG(E)	4	0.16	GGG(G)	27	0.75
***Hampala dispar* (AP011245)**											
Codon	Count	RSCU	Codon	Count	RSCU	Codon	Count	RSCU	Codon	Count	RSCU
UUU(F)	79	1.03	UCU(S)	53	0.98	UAU(Y)	93	1.18	UGU(C)	26	0.70
UUC(F)	75	0.97	UCC(S)	44	0.82	UAC(Y)	65	0.82	UGC(C)	48	1.30
UUA(L)	73	1.04	UCA(S)	47	0.87	UAA(*)	31	0.74	UGA(W)	74	1.25
UUG(L)	12	0.17	UCG(S)	8	0.15	UAG(*)	12	0.29	UGG(W)	44	0.75
CUU(L)	116	1.65	CCU(P)	109	1.41	CAU(H)	87	1.01	CGU(R)	29	0.89
CUC(L)	65	0.92	CCC(P)	59	0.76	CAC(H)	86	0.99	CGC(R)	43	1.31
CUA(L)	111	1.58	CCA(P)	120	1.55	CAA(Q)	72	1.64	CGA(R)	37	1.13
CUG(L)	45	0.64	CCG(P)	21	0.27	CAG(Q)	16	0.36	CGG(R)	22	0.67
AUU(I)	188	1.39	ACU(T)	139	1.55	AAU(N)	157	1.05	AGU(S)	59	1.10
AUC(I)	82	0.61	ACC(T)	76	0.85	AAC(N)	142	0.95	AGC(S)	112	2.08
AUA(M)	99	1.38	ACA(T)	121	1.35	AAA(K)	98	1.75	AGA(*)	47	1.13

AUG(M)	44	0.62	ACG(T)	22	0.25	AAG(K)	14	0.25	AGG(*)	77	1.84
GUU(V)	30	0.99	GCU(A)	34	0.95	GAU(D)	16	0.70	GGU(G)	31	0.89
GUC(V)	20	0.66	GCC(A)	52	1.45	GAC(D)	30	1.30	GGC(G)	24	0.69
GUA(V)	66	2.18	GCA(A)	56	1.57	GAA(E)	45	1.80	GGA(G)	63	1.81
GUG(V)	5	0.17	GCG(A)	1	0.03	GAG(E)	5	0.20	GGG(G)	21	0.60
Hampala salweenensis **(MW548258)**											
Codon	Count	RSCU	Codon	Count	RSCU	Codon	Count	RSCU	Codon	Count	RSCU
UUU(F)	86	1.11	UCU(S)	44	0.83	UAU(Y)	98	1.21	UGU(C)	27	0.71
UUC(F)	69	0.89	UCC(S)	45	0.84	UAC(Y)	64	0.79	UGC(C)	49	1.29
UUA(L)	63	0.89	UCA(S)	52	0.98	UAA(*)	30	0.70	UGA(W)	71	1.27
UUG(L)	18	0.25	UCG(S)	6	0.11	UAG(*)	17	0.40	UGG(W)	41	0.73
CUU(L)	115	1.62	CCU(P)	124	1.55	CAU(H)	85	0.98	CGU(R)	28	0.85
CUC(L)	64	0.90	CCC(P)	63	0.79	CAC(H)	89	1.02	CGC(R)	43	1.31
CUA(L)	131	1.85	CCA(P)	110	1.38	CAA(Q)	78	1.73	CGA(R)	35	1.07
CUG(L)	34	0.48	CCG(P)	23	0.29	CAG(Q)	12	0.27	CGG(R)	25	0.76
AUU(I)	176	1.36	ACU(T)	131	1.48	AAU(N)	152	1.04	AGU(S)	62	1.16
AUC(I)	82	0.64	ACC(T)	81	0.91	AAC(N)	141	0.96	AGC(S)	111	2.08
AUA(M)	100	1.37	ACA(T)	120	1.35	AAA(K)	93	1.77	AGA(*)	49	1.14
AUG(M)	46	0.63	ACG(T)	23	0.26	AAG(K)	12	0.23	AGG(*)	76	1.77
GUU(V)	38	1.24	GCU(A)	45	1.22	GAU(D)	14	0.60	GGU(G)	27	0.79
GUC(V)	19	0.62	GCC(A)	48	1.31	GAC(D)	33	1.40	GGC(G)	29	0.85
GUA(V)	60	1.95	GCA(A)	53	1.44	GAA(E)	42	1.62	GGA(G)	54	1.59
GUG(V)	6	0.20	GCG(A)	1	0.03	GAG(E)	10	0.38	GGG(G)	26	0.76
Hampala macrolepidota **(AP011186)**											
Codon	Count	RSCU	Codon	Count	RSCU	Codon	Count	RSCU	Codon	Count	RSCU
UUU(F)	65	0.92	UCU(S)	51	0.94	UAU(Y)	89	1.24	UGU(C)	29	0.75
UUC(F)	77	1.08	UCC(S)	38	0.70	UAC(Y)	54	0.76	UGC(C)	48	1.25
UUA(L)	64	0.90	UCA(S)	60	1.11	UAA(*)	30	0.77	UGA(W)	70	1.26
UUG(L)	21	0.30	UCG(S)	8	0.15	UAG(*)	14	0.36	UGG(W)	41	0.74
CUU(L)	111	1.57	CCU(P)	118	1.52	CAU(H)	93	0.97	CGU(R)	20	0.57
CUC(L)	70	0.99	CCC(P)	73	0.94	CAC(H)	99	1.03	CGC(R)	50	1.42
CUA(L)	117	1.65	CCA(P)	101	1.30	CAA(Q)	79	1.68	CGA(R)	43	1.22
CUG(L)	42	0.59	CCG(P)	19	0.24	CAG(Q)	15	0.32	CGG(R)	28	0.79

AUU(I)	189	1.38	ACU(T)	131	1.47	AAU(N)	152	1.03	AGU(S)	59	1.09
AUC(I)	84	0.62	ACC(T)	81	0.91	AAC(N)	142	0.97	AGC(S)	109	2.01
AUA(M)	97	1.34	ACA(T)	119	1.34	AAA(K)	96	1.78	AGA(*)	41	1.05
AUG(M)	48	0.66	ACG(T)	25	0.28	AAG(K)	12	0.22	AGG(*)	71	1.82
GUU(V)	28	0.94	GCU(A)	34	0.94	GAU(D)	14	0.61	GGU(G)	28	0.77
GUC(V)	22	0.74	GCC(A)	60	1.66	GAC(D)	32	1.39	GGC(G)	31	0.86
GUA(V)	63	2.12	GCA(A)	47	1.30	GAA(E)	45	1.73	GGA(G)	59	1.63
GUG(V)	6	0.20	GCG(A)	4	0.11	GAG(E)	7	0.27	GGG(G)	27	0.74
***Hampala macrolepidota* (KF670818)**											
Codon	Count	RSCU	Codon	Count	RSCU	Codon	Count	RSCU	Codon	Count	RSCU
UUU(F)	66	0.92	UCU(S)	50	0.93	UAU(Y)	90	1.26	UGU(C)	29	0.75
UUC(F)	77	1.08	UCC(S)	37	0.69	UAC(Y)	53	0.74	UGC(C)	48	1.25
UUA(L)	64	0.91	UCA(S)	60	1.11	UAA(*)	31	0.78	UGA(W)	69	1.25
UUG(L)	20	0.28	UCG(S)	8	0.15	UAG(*)	14	0.35	UGG(W)	41	0.75
CUU(L)	109	1.54	CCU(P)	121	1.55	CAU(H)	92	0.97	CGU(R)	21	0.59
CUC(L)	70	0.99	CCC(P)	70	0.89	CAC(H)	98	1.03	CGC(R)	51	1.44
CUA(L)	119	1.68	CCA(P)	102	1.30	CAA(Q)	79	1.68	CGA(R)	43	1.21
CUG(L)	42	0.59	CCG(P)	20	0.26	CAG(Q)	15	0.32	CGG(R)	27	0.76
AUU(I)	189	1.38	ACU(T)	132	1.49	AAU(N)	153	1.04	AGU(S)	59	1.10
AUC(I)	85	0.62	ACC(T)	80	0.90	AAC(N)	142	0.96	AGC(S)	109	2.02
AUA(M)	96	1.32	ACA(T)	118	1.33	AAA(K)	96	1.78	AGA(*)	42	1.06
AUG(M)	49	0.68	ACG(T)	25	0.28	AAG(K)	12	0.22	AGG(*)	72	1.81
GUU(V)	30	1.00	GCU(A)	34	0.94	GAU(D)	13	0.58	GGU(G)	27	0.75
GUC(V)	21	0.70	GCC(A)	60	1.66	GAC(D)	32	1.42	GGC(G)	31	0.86
GUA(V)	63	2.10	GCA(A)	47	1.30	GAA(E)	45	1.73	GGA(G)	59	1.64
GUG(V)	6	0.20	GCG(A)	4	0.11	GAG(E)	7	0.27	GGG(G)	27	0.75

Table S7. The RSCU abundance and CDsPT calculation of the complete PCGs of *Hampala* mitogenomes.

**Amino Acid**	***H. macrolepidota* (PP937078)**		***H. macrolepidota* (AP011186)**		***H. macrolepidota* (KF670818)**		***H. dispar* (AP011245)**		***H. salweenensis* (MW548258)**	
	**Abundance**	**CDsPT**	**Abundance**	**CDsPT**	**Abundance**	**CDsPT**	**Abundance**	**CDsPT**	**Abundance**	**CDsPT**
Ala (A)	140	36.84	145	38.16	145	38.15	143	37.65	147	38.69
Cys (C)	69	18.16	77	20.26	77	20.26	74	19.48	76	20.01
Ile (I)	268	70.53	273	71.84	274	72.09	270	71.09	258	67.91
Leu (L)	425	111.84	425	111.84	424	111.55	422	111.11	425	111.87
Met (M)	145	38.16	145	38.16	145	38.15	143	37.65	146	38.43
Phe (F)	147	38.68	142	37.37	143	37.62	154	40.55	155	40.80
Trp (W)	109	28.68	111	29.21	110	28.94	118	31.07	112	29.48
Val (V)	124	32.63	119	31.32	120	31.57	121	31.86	123	32.38
Arg (R)	145	38.16	141	37.11	142	37.36	131	34.49	131	34.48
Asn (N)	291	76.58	294	77.37	295	77.61	299	78.73	293	77.13
Asp (D)	47	12.37	46	12.11	45	11.84	46	12.11	47	12.37
Gln (Q)	96	25.26	94	24.74	94	24.73	88	23.17	90	23.69
Glu (E)	50	13.16	52	13.68	52	13.68	50	13.16	52	13.69
Lys (K)	109	28.68	108	28.42	108	28.41	112	29.49	105	27.64
Gly (G)	144	37.89	145	38.16	144	37.88	139	36.60	136	35.80
His (H)	188	49.47	192	50.53	190	49.99	173	45.55	174	45.80
Pro (P)	311	81.84	311	81.84	313	82.35	309	81.36	320	84.23
Ser (S)	326	85.79	325	85.53	323	84.98	323	85.04	320	84.23
Thr (T)	362	95.26	356	93.68	355	93.40	358	94.26	355	93.45
Tyr (Y)	147	38.68	143	37.63	143	37.62	158	41.60	162	42.64
Stop	157	41.32	156	41.05	159	41.83	167	43.97	172	45.28

Table S8. Detailed comparison of anticodons found in the tRNA genes within the mitogenome of three *Hampala* species (five sequences).

**tRNA Genes**	***H. macrolepidota*** **(PP937078)**	***H. salweenensis*** **(MW548258)**	***H. dispar*** **(AP011245)**	***H. macrolepidota*** **(KF670818)**	***H. macrolepidota*** **(AP011186)**
tRNA-Phe (F)	GAA	GAA	GAA	GAA	GAA
tRNA-Val (V)	TAC	TAC	TAC	TAC	TAC
tRNA-Leu (L2)	TAA	TAA	TAA	TAA	TAA
tRNA-Ile (I)	GAT	GAT	GAT	GAT	GAT
tRNA-Gln (Q)	TTG	TTG	TTG	TTG	TTG
tRNA-Met (M)	CAT	CAT	CAT	CAT	CAT
tRNA-Trp (W)	TCA	TCA	TCA	TCA	TCA
tRNA-Ala (A)	TGC	TGC	TGC	TGC	TGC
tRNA-Asn (N)	GTT	GTT	GTT	GTT	GTT
tRNA-Cys (C)	GCA	GCA	GCA	GCA	GCA
tRNA-Tyr (Y)	GTA	GTA	GTA	GTA	GTA
tRNA-Ser (S2)	TGA	TGA	TGA	TGA	TGA
tRNA-Asp (D)	GTC	GTC	GTC	GTC	GTC
tRNA-Lys (K)	TTT	TTT	TTT	TTT	TTT
tRNA-Gly (G)	TCC	TCC	TCC	TCC	TCC
tRNA-Arg (R)	TCG	TCG	TCG	TCG	TCG
tRNA-His (H)	GTG	GTG	GTG	GTG	GTG
tRNA-Ser (S1)	GCT	GCT	GCT	GCT	GCT
tRNA-Leu (L1)	TAG	TAG	TAG	TAG	TAG
tRNA-Glu (E)	TTC	TTC	TTC	TTC	TTC
tRNA-Thr (T)	TGT	TGT	TGT	TGT	TGT
tRNA-Pro (P)	TGG	TGG	TGG	TGG	TGG

Table S9. The mean intra- and inter-species genetic distance among *Hampala* species based on the K2P model estimated from concatenated 13 PCGs sequences.

**Species**	***H. macrolepidota* (PP937078)**	***H. macrolepidota* (AP011186)**	***H. macrolepidota* (KF670818)**	***H. dispar* (AP011245)**	***H. salweenensis* (MW548258)**
H. macrolepidota (PP937078)					
H. macrolepidota (AP011186)	0.02742				
H. macrolepidota (KF670818)	0.02761	0.00246			
H. dispar (AP011245)	0.09629	0.09757	0.09763		
H. salweenensis (MW548258)	0.10228	0.10271	0.10254	0.07562	

Table S10. The mean inter-population genetic distance within *H. macrolepidota* across Asian region based on the K2P model estimated from *COI* and *Cytb* partial gene sequences.

Gene	Locality	Inter-						
		Indonesia	Singapore	Malaysia	Vietnam	China	Unknown	
COI	Indonesia							
	Singapore	0.351						
	Malaysia	0.429	0.194					
	Vietnam	0.642	0.290	0.485				
	China	4.133	3.918	3.918	4.220			
	Unknown	4.133	3.918	3.918	4.220	0.000		
Cytb	Locality	Indonesia	Malaysia	Vietnam	Laos	China	Unknown	Thailand
	Indonesia							
	Malaysia	2.287						
	Vietnam	2.877	4.460					
	Laos	3.207	4.156	0.313				
	China	3.207	4.156	0.313	0.000			
	Unknown	3.373	4.325	0.470	0.156	0.156		
	Thailand	3.406	4.358	0.501	0.188	0.188	0.344	

Table 11. The haplotype composition of *H. macrolepidota* inferred from partial mitochondrial *COI* gene sequences obtained in this study and retrieved from the GenBank database. '*n*' indicates the number of sequences analyzed.

Haplotype	*n*	Accession Number and Locality
Hap_1	1	PQ735333_Indonesia
Hap_2	1	PP937078_Indonesia
Hap_3	13	PQ735426_Indonesia PQ735525_Indonesia PQ735588_Indonesia PQ735601_Indonesia PQ735606_Indonesia PQ735744_Indonesia PQ735880_Indonesia KU692540_Indonesia KU692541_Indonesia KU692543_Indonesia HM156390_Malaysia MN342602_Singapore MN342603_Singapore
Hap_4	20	PQ735441_Indonesia PQ735454_Indonesia PQ735474_Indonesia PQ735519_Indonesia PQ735667_Indonesia PQ735849_Indonesia KM213069_Indonesia KM213070_Indonesia KM213071_Indonesia KM213072_Indonesia KM213073_Indonesia KM213074_Indonesia KM213075_Indonesia KM213076_Indonesia KM213077_Indonesia KM213078_Indonesia KM213079_Indonesia KM213080_Indonesia MZ643248_Indonesia JF781171_Malaysia
Hap_5	2	PQ735538_Indonesia PQ735860_Indonesia
Hap_6	1	PQ735724_Indonesia
Hap_7	1	KU692542_Indonesia
Hap_8	1	JF781170_Malaysia
Hap_9	1	MK116339_Vietnam
Hap_10	4	HQ236005_China HM536886_China AP011186_Unknown KF670818_Unknown

Table 12. The haplotype composition of *H. macrolepidota* inferred from partial mitochondrial *Cytb* gene sequences obtained in this study and retrieved from the GenBank database. '*n*' indicates the number of sequences analyzed.

Haplotype	*n*	Accession Number and Locality
Hap_1	1	PP937078_Indonesia
Hap_2	44	AY697301_Malaysia AY697302_Malaysia AY697303_Malaysia AY697304_Malaysia AY697305_Malaysia AY697307_Malaysia AY697309_Malaysia AY697311_Malaysia AY697312_Malaysia AY697313_Malaysia AY697314_Malaysia AY697315_Malaysia AY697319_Malaysia AY697320_Malaysia AY697321_Malaysia AY697322_Malaysia AY697323_Malaysia AY697324_Malaysia AY697325_Malaysia AY697326_Malaysia AY697327_Malaysia AY697328_Malaysia AY697329_Malaysia AY697330_Malaysia AY697331_Malaysia AY697332_Malaysia AY697343_Malaysia AY697344_Malaysia AY697345_Malaysia AY697346_Malaysia AY697347_Malaysia AY697348_Malaysia AY697349_Malaysia AY360419_Malaysia AY360425_Malaysia AY360426_Malaysia AY360427_Malaysia AY360428_Malaysia AY360429_Malaysia AY360430_Malaysia AY360431_Malaysia AY527072_Malaysia AY527073_Malaysia AY243351_Malaysia
Hap_3	10	AY697306_Malaysia AY697308_Malaysia AY697310_Malaysia AY697316_Malaysia AY697317_Malaysia AY697318_Malaysia AY697350_Malaysia AY360415_Malaysia AY360417_Malaysia AY360412_Malaysia
Hap_4	1	AP011186_Unknown
Hap_5	8	KF670818_Unknown OQ561428_Thailand OQ561429_Thailand JQ346142_Laos MH688236_Laos MH688237_Laos KC696545_China HM536790_China
Hap_6	1	AY697333_Malaysia
Hap_7	5	AY697334_Malaysia AY697335_Malaysia AY697339_Malaysia AY360420_Malaysia AY360421_Malaysia
Hap_8	8	AY697336_Malaysia AY697337_Malaysia AY697338_Malaysia AY697340_Malaysia AY697341_Malaysia AY697342_Malaysia AY360422_Malaysia AY360423_Malaysia
Hap_9	7	AY697351_Malaysia AY697352_Malaysia AY697354_Malaysia AY697356_Malaysia AY527075_Malaysia AY527077_Malaysia AY527079_Malaysia
Hap_10	4	AY697353_Malaysia AY697355_Malaysia AY527076_Malaysia AY527078_Malaysia
Hap_11	3	AY697357_Malaysia AY697358_Malaysia AY697359_Malaysia
Hap_12	2	AY360416_Malaysia AY243352_Malaysia
Hap_13	1	AY360418_Malaysia
Hap_14	1	AY360424_Malaysia
Hap_15	1	AY527066_Malaysia
Hap_16	1	AY527067_Malaysia
Hap_17	1	AY527068_Malaysia
Hap_18	1	AY527069_Malaysia
Hap_19	1	AY527070_Malaysia
Hap_20	1	AY527071_Malaysia
Hap_21	1	AY527074_Malaysia
Hap_22	4	AY445067_Malaysia AY445068_Malaysia AY445069_Malaysia AY445071_Malaysia
Hap_23	1	AY445070_Malaysia
Hap_24	1	AY360434_Malaysia
Hap_25	3	OQ561430_Thailand OQ561431_Thailand OQ561432_Thailand
Hap_26	1	DQ464974_Vietnam

References

Behera, B.K.; Rout, A.K.; Baisvar, V.S.; Paria, P.; Singh, N.S.; Ghadei, S.S.; Jana, A.K.; Parida, P.K.; Das, B.K. The complete mitochondrial genome sequence of *Osteobrama belangeri* (Cyprinidae) and its comparison with other related Cypriniformes fish species. *Mitochondrial DNA Part B* **2019**, *4*, 2330–2331.

Biswal, J.R.; Singh, R.K.; Dutta, N.; Pathak, A.; Lal, K.K.; Mohindra, V.; Sah, R.S.; Jena, J.K. The complete mitochondrial genome of olive barb, *Systomus sarana sarana* (Hamilton, 1822) and its phylogenetic status. *Mitochondrial DNA Part B* **2017**, *2*, 940–942.

Collins, R. A.; Armstrong, K. F.; Meier, R.; Yi, Y.; Brown, S. D. J.; Cruickshank, R. H.; Keeling, S.; Johnston, C. Barcoding and border biosecurity: Identifying cyprinid fishes in the aquarium trade. *PLoS ONE* **2012**, 7, e28381.

Dahruddin, H.; Hutama, A.; Busson, F.; Sauri, S.; Hanner, R.; Keith, P.; Hadiaty, R.; Hubert, N. Revisiting the ichthyodiversity of Java and Bali through DNA barcodes: Taxonomic coverage, identification accuracy, cryptic diversity, and identification of exotic species. *Mol. Ecol. Resour*. **2017**, *17*, 288–299.

Delrieu-Trottin, E.; Ben Chéhida, S.; Sukmono, T.; Dahruddin, H.; Sholihah, A.; Kustiati, K.; Fitriana, Y.; Muchlisin, Z.A.; Elvyra, R.; Wibowo, A.; Utama, I.V.; Nurhaman, U.; Sauri, S.; Risdawati, R.; Zein, M.S.A.; Pouzadoux, J.; Agnèse, J.-F.; Tilak, M.K.; Page, L.M.; von Rintelen, T.; Wowor, D.; Steinke, D.; Mona, S.; Rüber, L.; Hebert, P.D.N.; Hubert, N. Aquatic biotas of Sundaland are fragmented but not refugial. *Syst. Biol*. **2025**, *74*, 685–699.

Esa, Y.B.; Japning, J.R.R.; Rahim, K.A.A.; Siraj, S.S.; Daud, S.K.; Tan, S.G.; Sungan, S. Phylogenetic relationships among several freshwater fishes (Family: Cyprinidae) in Malaysia inferred from partial sequencing of the Cytochrome b mitochondrial DNA gene. *Pertanika J. Trop. Agric. Sci*. **2012**, *35*, 307–318.

Gillet, B.; Cottet, M.; Destanque, T.; Kue, K.; Descloux, S.; Chanudet, V.; Hughes, S. Direct fishing and eDNA metabarcoding for biomonitoring during a 3-year survey significantly improves number of fish detected around a Southeast Asian reservoir. *PLoS ONE* **2018**, *13*, e0208592.

Jang-Liaw, N.-H.; Chang, C.-H.; Tsai, C.-L. Complete mitogenomes of two *Puntius* in Taiwan: *P. semifasciolatus* and *P. snyderi* (Cypriniformes: Cyprinidae). *Mitochondrial DNA* **2013**, *24*, 228–230.

Kundu, S.; Binarao, J.D.; De Alwis, P.S.; Kim, A.R.; Lee, S.-R.; Andriyono, S.; Gietbong, F.Z.; Kim, H.-W. First mitogenome of endangered *Enteromius thysi* (Actinopterygii: Cypriniformes: Cyprinidae) from Africa: Characterization and phylogeny. *Fishes* **2023**, *8*, 25.

Liu, M.; Huang, F.; Liu, S. The mitochondrial genome of *Hampala macrolepidota* (Cypriniformes, Cyprinidae). *Mitochondrial DNA* **2015**, *26*, 807–808.

Makmur, S.; Arfiati, D.; Bintoro, G.; Ekawati, A. W. Morphological, meristic characteristics, and mtDNA analysis of *Hampala* fish (*Hampala macrolepidota* Kuhl & Van Hasselt 1823) from Ranau Lake, Indonesia. *J. Biodivers. Environ. Sci*. **2014**, *5*, 447–455.

Pan, Y.; Xiang, X.; Tong, Y.; Hu, S.; Zhou, D.; Wu, G.; Qin, Y. The complete mitochondrial genome of *Pethia padamya* (Actinopteri, Cyprinidae). *Mitochondrial DNA Part B* **2023**, *8*, 426–429.

Panprommin, D.; Soontornprasit, K.; Tuncharoen, S.; Pithakpol, S.; Kannika, K.; Wongta, K. DNA barcoding for fish species identification and diversity assessment in the Mae Tam reservoir, Thailand. *Fish. Aquat. Sci*. **2023**, *26*, 548–557.

Pasco-Viel, E.; Veran, M.; Viriot, L. Bleeker was right: Revision of the genus *Cyclocheilichthys* (Bleeker 1859) and resurrection of the genus *Anematichthys* (Bleeker 1859), based on morphological and molecular data of Southeast Asian Cyprininae (Teleostei, Cypriniformes). *Zootaxa* **2012**, *3586*, 41–54.

Qiao, Z.; Xing, J.; Li, F. Structural analysis and phylogenetic relationships of a teleost fish, *Pethia stoliczkana* based on the complete mitochondrial genome sequence. *Pak. J. Zool*. **2024**, *56*, 853–860.

Ryan, J.R.J.; Esa, Y.B. Phylogenetic analysis of *Hampala* fishes (Subfamily Cyprininae) in Malaysia inferred from partial mitochondrial Cytochrome b DNA sequences. *Zool. Sci*. **2006**, *23*, 893–901.

Saitoh, K.; Sado, T.; Mayden, R.L.; Hanzawa, N.; Nakamura, K.; Nishida, M.; Miya, M. Mitogenomic evolution and interrelationships of the Cypriniformes (Actinopterygii: Ostariophysi): The first evidence toward resolution of higher-level relationships of the world's largest freshwater fish clade based on 59 whole mitogenome sequences. *J. Mol. Evol*. **2006**, *63*, 826–841.

Schedel, F.D.B.; Musilova, Z.; Indermaur, A.; Bitja-Nyom, A.R.; Salzburger, W.; Schliewen, U.K. Towards the phylogenetic placement of the enigmatic African genus *Prolabeops* Schultz, 1941. *J. Fish Biol*. **2022**, *101*, 1333–1342.

Song, L.M.; Munian, K.; Abd Rashid, Z.; Bhassu, S. Characterisation of Asian snakehead murrel *Channa striata* (Channidae) in Malaysia: An insight into molecular data and morphological approach. *Sci. World J*. **2013**, *12*, 917506.

Sun, C.-H.; Sun, P.-Y.; Lao, Y.-L.; Wu, T.; Zhang, Y.-N.; Huang, Q.; Zhang, Q. Mitogenome of a monotypic genus, *Oliotius* Kottelat, 2013 (Cypriniformes: Cyprinidae): Genomic characterization and phylogenetic position. *Gene* **2023**, *851*, 147035.

Thai, B.T.; Si, V.N.; Phan, P.D.; Austin, C.M. Phylogenetic evaluation of subfamily classification of the Cyprinidae focusing on Vietnamese species. *Aquat. Living Resour*. **2007**, *20*, 143–153.

Vu, Q.D.H.; Truong, O.T.; Linh, T.T.; Dang, B.T. Molecular phylogeny of Cyprinidae and Cobitidae (Teleostei: Cypriniformes)—Implication for Mekong fish. Proc. ISER 169th Int. Conf. 2018, 16 pp.

Wang, J.; Wu, X.; Chen, Z.; Yue, Z.; Ma, W.; Chen, S.; Xiao, H.; Murphy, R.W.; Zhang, Y.; Zan, R.; Luo, J. Molecular phylogeny of European and African *Barbus* and their West Asian relatives in the Cyprininae (Teleostei: Cypriniformes) and orogenesis of the Qinghai-Tibetan Plateau. *Chin. Sci. Bull*. **2013**, *58*, 3738–3746.

Wang, X.; Gan, X.; Li, J.; Chen, Y.; He, S. Cyprininae phylogeny revealed independent origins of the Tibetan Plateau endemic polyploid cyprinids and their diversifications related to the Neogene uplift of the plateau. *Sci. China Life Sci.* **2016**, 59, 1149–1165.

Wulandari, T. N. M. Fish identification based on partial fragments of the mitochondrial COI subunit I gene. *E3S Web of Conferences* **2021**, *322*, 01038.

Xu, R.; Zhao, Z.X.; Zhang, Y.; Xu, P.; Sun, X.W. Complete mitochondrial genome of rosy barb, *Puntius conchonius*. *Mitochondrial DNA* **2015**, *26*, 955–956.

Yang, L.; Mayden, R.L.; Sado, T.; He, S.; Saitoh, K.; Miya, M. Molecular phylogeny of the fishes traditionally referred to Cyprinini sensu stricto (Teleostei: Cypriniformes). *Zool. Scr*. **2010**, *39*, 527–550.
